# Comparative iron oxide nanoparticle cellular dosimetry and response in mice by the inhalation and liquid cell culture exposure routes

**DOI:** 10.1186/s12989-014-0046-4

**Published:** 2014-09-30

**Authors:** Justin G Teeguarden, Vladimir B Mikheev, Kevin R Minard, William C Forsythe, Wei Wang, Gaurav Sharma, Norman Karin, Susan C Tilton, Katrina M Waters, Bahman Asgharian, Owen R Price, Joel G Pounds, Brian D Thrall

**Affiliations:** Pacific Northwest National Laboratory, Richland, WA 99352 USA; Battelle Memorial Institute, 505 King Avenue, Columbus, OH 43201-2696 USA; Oak Ridge National Laboratory, P.O. Box 2008, Oak Ridge, TN 37831 USA; Roswell Park Cancer Institute, Elm & Carlton Streets, Buffalo, NY 14263 USA; Applied Research Associates, 8537 Six Forks Road, Raleigh, NC 27615-2963 USA

**Keywords:** Magnetic particle detection, Nano-aerosol, In vivo testing, Lung deposition, Nanoparticle, Dosimetry, Extrapolation

## Abstract

**Background:**

Toxicity testing the rapidly growing number of nanomaterials requires large scale use of in vitro systems under the presumption that these systems are sufficiently predictive or descriptive of responses in in vivo systems for effective use in hazard ranking. We hypothesized that improved relationships between in vitro and in vivo models of experimental toxicology for nanomaterials would result from placing response data in vitro and in vivo on the same dose scale, the amount of material associated with cells.

**Methods:**

Balb/c mice were exposed nose-only to an aerosol (68.6 nm CMD, 19.9 mg/m^3^, 4 hours) generated from of 12.8 nm superparamagnetic iron oxide particles (SPIO). Target cell doses were calculated, histological evaluations conducted, and biomarkers of response were identified by global transcriptomics. Representative murine epithelial and macrophage cell types were exposed in vitro to the same material in liquid suspension for four hours and levels of nanoparticle regulated cytokine transcripts identified in vivo were quantified as a function of measured nanoparticle cellular dose.

**Results:**

Target tissue doses of 0.009-0.4 μg SPIO/cm^2^ in lung led to an inflammatory response in the alveolar region characterized by interstitial inflammation and macrophage infiltration. In vitro, higher target tissue doses of ~1.2-4 μg SPIO/ cm^2^ of cells were required to induce transcriptional regulation of markers of inflammation, CXCL2 & CCL3, in C10 lung epithelial cells. Estimated in vivo macrophage SPIO nanoparticle doses ranged from 1-100 pg/cell, and induction of inflammatory markers was observed in vitro in macrophages at doses of 8-35 pg/cell.

**Conclusions:**

Application of target tissue dosimetry revealed good correspondence between target cell doses triggering inflammatory processes in vitro and in vivo in the alveolar macrophage population, but not in the epithelial cells of the alveolar region. These findings demonstrate the potential for target tissue dosimetry to enable the more quantitative comparison of in vitro and in vivo systems and advance their use for hazard assessment and extrapolation to humans. The mildly inflammogentic cellular doses experienced by mice were similar to those calculated for humans exposed to the same material at the existing permissible exposure limit of 10 mg/m^3^ iron oxide (as Fe).

**Electronic supplementary material:**

The online version of this article (doi:10.1186/s12989-014-0046-4) contains supplementary material, which is available to authorized users.

## Background

The field of in vitro toxicology continues its long and consistent evolution from an exploratory platform used principally for mechanistic studies to one suited for meeting multiple emerging and rapidly growing toxicity testing needs. There are growing scientific and regulatory dependencies on the ability of these systems to sufficiently recapitulate the biology of full organisms so they can be used effectively as tools not only for classification of compounds by mode of action, but for more quantitative applications such as hazard ranking and risk assessment [1]. The U.S. EPA and the National Toxicology Program [2] have established large, inter- and intra—agency collaborative research programs to pioneer in vitro based ranking and risk assessment methodologies. These efforts initially focused on organic and inorganic chemicals to leverage the decades of toxicological testing experience with these materials and the large amount of information available on mode of action, biomarkers, structure activity relationships, in vivo and epidemiological data. Nanomaterials, with unique physical, chemical and biological properties but a very limited toxicological data base, now face the need for similar testing [3].

Nanomaterials comprise an immense class of particles with medical, environmental and commercially important properties not found in the elements or bulk materials they are composed of. Their small size (traditionally ≤ 100 nm in at least one dimension) impart unique physicochemical, conductive, catalytic and biological properties, among others [4]. Market research predicts a global market of one trillion dollars a year [5], and an almost limitless member class can be expected given the multiple characteristics that create unique materials (e.g. size, shape, composition, crystallinity, etc). By public health standards, nanomaterials are toxicologically new, representing a largely understudied group of materials. Testing of the scale required to address the public heath aspects of nanomaterial production and use is universally understood to rely heavily on cost-effective in vitro systems. Proof of the suitability of in vitro systems for predicting in vivo responses will be obligatory when quantitative predictions from these systems, for example in hazard screening and dose-incidence data, are primary objectives.

Sayes et al. were the first to attempt coordinated in vitro and in vivo studies on nanoparticles to explore the correlation between findings in these two test systems. Inflammatory responses to crystalline and amorphous silica and zinc oxide nanoparticles in male rats exposed by intratracheal instillation (IT) to 1 or 5 mg/kg were compared to those from rat macrophages and epithelial cells exposed in vitro to nanoparticle concentrations of 0.0052-520 μg/cm^2^ [6]. Neither this study nor the follow-on study by the same authors [7] focusing on C60 fullerenes, found correlations between in vitro and in vivo responses to nanoparticles. Considering the earlier work by Seagrave et al. [8], which reported no correspondence between in vitro and in vivo toxicity of diesel exhaust particulates and soluble extracts, in vitro systems, as they are currently used, have considerable limitations for assessment of in vivo toxicity of particulates.

The Sayes and Seagrove studies used the conventional approach of comparing response data incidence in each system to particle exposure; liquid concentrations or air concentration in vitro, and total dose (mass, or mass/kg) for the IT or inhalation studies. Without a common measure of the amount of nanoparticles (or diesel particulates) in contact with the cells—target cell dose—in each, the magnitude of response cannot be accurately placed on the dose axis for purposes of comparing toxicities. This particular limitation, common to nanotoxicity testing, is likely to be a major factor contributing to the low rates of correspondence between in vitro and in vivo responses to these materials and a barrier to deeper understanding the root causes. Biological responses to nanoparticles in vitro may correlate well to the corresponding effects in vivo, but with dose-dependencies in the nature of the effects (mode of action), the magnitude of effects, the timing of the effects and the potential saturation of key processes, these relationships are obscured when nominal exposures (μg/ml) are used as a dose-metric. It is plausible that broader implementation of the target tissue dosimetry paradigm common to the field of chemical risk assessment will improve correlations and help advance the use of in vitro systems to predict nanomaterial hazard and risk.

We hypothesized that improved relationships between in vitro and in vivo models of experimental toxicology for nanomaterials would result from placing response data in vitro and in vivo on the same dose scale, the amount of material associated with cells (target cell dose). The objective of the work was to test this hypothesis in the Balb/c mouse, a common test animal, by conducting coordinated in vitro and in vivo inhalation toxicity studies using cell types representing the mouse target tissue, in this case the lung. Superparamagnetic iron oxide (SPIO) nanoparticles were selected as a proxy for low-solubility metal oxides, as well as for their commercial and medical importance [9,10], and the availability of a novel analytical platform that allows rapid, quantification of cellular levels of SPIO nanoparticles [11]. To our knowledge, this is the first study that directly tests the comparability of in vitro and in vitro responses to a nanomaterial by anchoring response data to target cell dosimetry.

## Results

### SPIO nanoparticle characterization

#### As produced particles

TEM images of the synthesized Fe_3_O_4_ nanoparticles are shown in Additional file 1. The average size of the nanoparticles was 12.8 nm by SEM analysis (Additional file 1). High-resolution TEM analysis confirmed the SPIO particles had the crystal structure of magnetite (Additional file 1). Because of their small size, the Fe_3_O_4_ nanoparticles were superparamagnetic. Surface modification with COO^−^ groups doubled the zeta potential to -41 mV and improved their dispersity in water (Additional file 2). Given a density of 5.2 grams/cm^3^, a picogram of SPIO particles would contain 154,015 primary 13 nm diameter particles with a surface area of 8.87 × 10^−7^ cm^2^.

#### In vivo particle exposure characteristics

**Figure 1:**
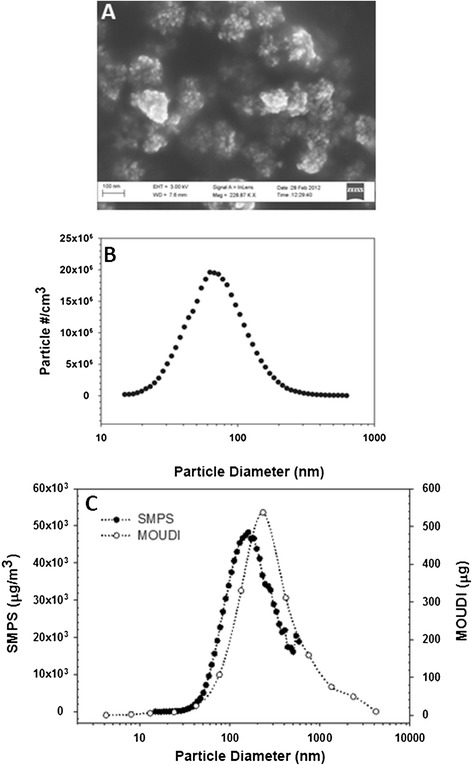
**Particle size distributions. A)** SEM image of SPIO nanoparticle agglomerates taken from MOUDI stage #7 (ECD = 233 nm); **B)** SMPS particle size distribution used to determine the CMD; **C)** Mass-based particle size distribution measured using nano-MOUDI (μg) and derived from SMPS (μg/m^3^). The MMD was 207 nm measured by nano-MOUDI and 171 nm when derived from SMPS data using a particle density of 5 g/cm^3^.

SPIO nanoparticle aerosol concentrations were uniformly distributed across the exposure carousel with little variation between (3.1% relative standard deviation (RSD)) and within (3.3% RSD) the carousel tiers (Additional file 3). Measured each hour (Scanning mobility particle sizer (SPMS), gravimetric filter analysis and nano micro-orifice uniform-deposit impactor (MOUDI)), SPIO nanoparticle aerosol concentrations were also stable and consistent across the full duration of the four hour inhalation experiment. The average particle number concentration measured by SMPS was 11 × 10^6^ particles/cm^3^, with an RSD of 20.7% across the four measurement periods. The average mass concentration collected on the filter was 19.9 mg/m^3^ (19.9 ng/cm^3^) with an RSD of 11.1% across the four measurement periods (Additional file 3). Particle size distributions were determined concomitantly with aerosol concentration measurements using SMPS count median diameter (CMD). MOUDI mass median diameter (MMD) data were also collected and then compared with SMPS (MMD) data. Particle size was stable with minimal variation through the duration of exposure period (Additional file 3). The average CMD was 68.6 nm (mean GSD 1.6). Particle MMD (MOUDI impactor) was 207 nm (GSD, 2.5) comparing well to particle MMD obtained from SMPS data (calculated from CMD at particle density 5 g/cm^3^), which was 171 nm (GSD, 1.8) (Figure 1). The SMPS upper size limit was restricted by ~1 μm whereas MOUDI upper range reaches ~10 μm. SEM imaging of MOUDI Stage #7(effective cut-off diameter of 233 nm), shows an accumulation of approximately 100 nm agglomerates of the 12.8 nm SPIO particles (Figure 1).

Mice were exposed to 19.9 mg/m^3^ of SPIO nanoparticles for 4 hours. The Occupational Safety and Health Association (OSHA) Permissible Exposure Limit (PEL) for iron oxide particles is 10 mg/m^3^ (as Fe), or 14 mg/m^3^ as Fe_2_O_3_. Our time weighted average (TWA) external exposure in mice was 4 hr × 19.9 mg/m^3^ (80 mg*hr/m^3^), while the PEL would allow 8 hr × 14 mg/m^3^ (112 mg*hr/m^3^). These comparisons do not address the role of particle clearance in the two species.

### Mouse respiratory physiology during SPIO exposures

Relative humidity and temperature at the nose port were nearly constant throughout the study. Average nose-port temperature for the sham (control) unit was 23.4°C (SD = 0.2) and average RH was 40% (SD = 1). Average nose-port temperature for the SPIO nanoparticle exposed unit was 24.3°C (SD = 0.1) and average RH was 46% (SD = 5). The tidal volume (TV, Additional file 4), respiratory rate (RR, Additional file 5), and minute ventilation (MV, Additional file 6) were measured for both control and SPIO nanoparticle exposed groups of animals, with minimal differences in the parameters. For the SPIO nanoparticle exposed group, the RR, TV, and MV were 371 (SD 29) breaths/min, 0.24 (SD 0.02) ml/breath and 77 (SD 8) ml/minute, respectively.

### SPIO Nanoparticle lung deposition, regional dosimetry and clearance

**Figure 2:**
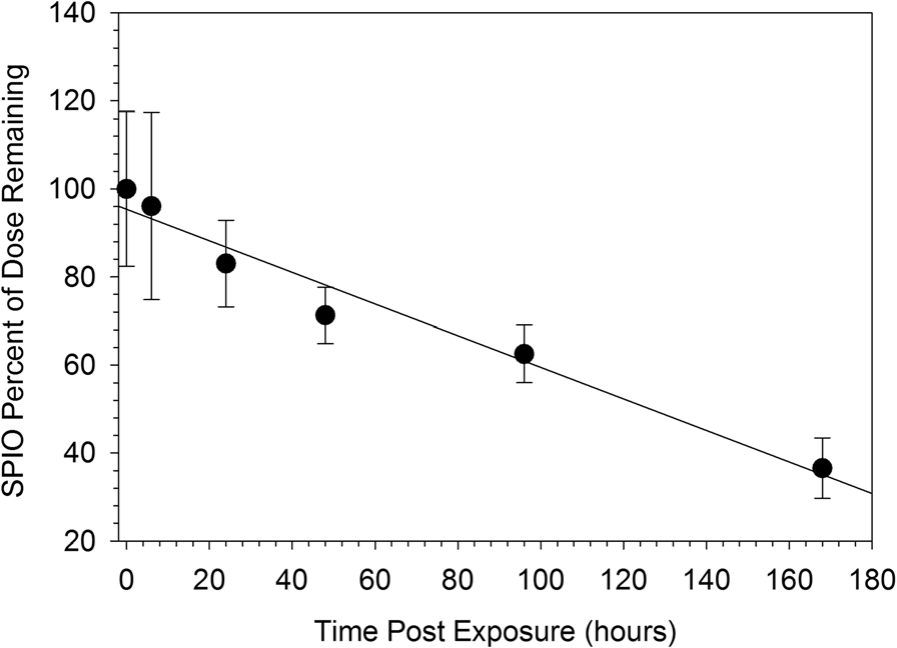
**Total deposited dose and clearance of SPIO nanoparticles from the lungs of Balb/c mice over a one week post exposure period.** Clearance was linear, with a single phase, corresponding to a rate of ~8.6% of deposited dose per day or 0.05 μg/hour.

**Figure 3:**
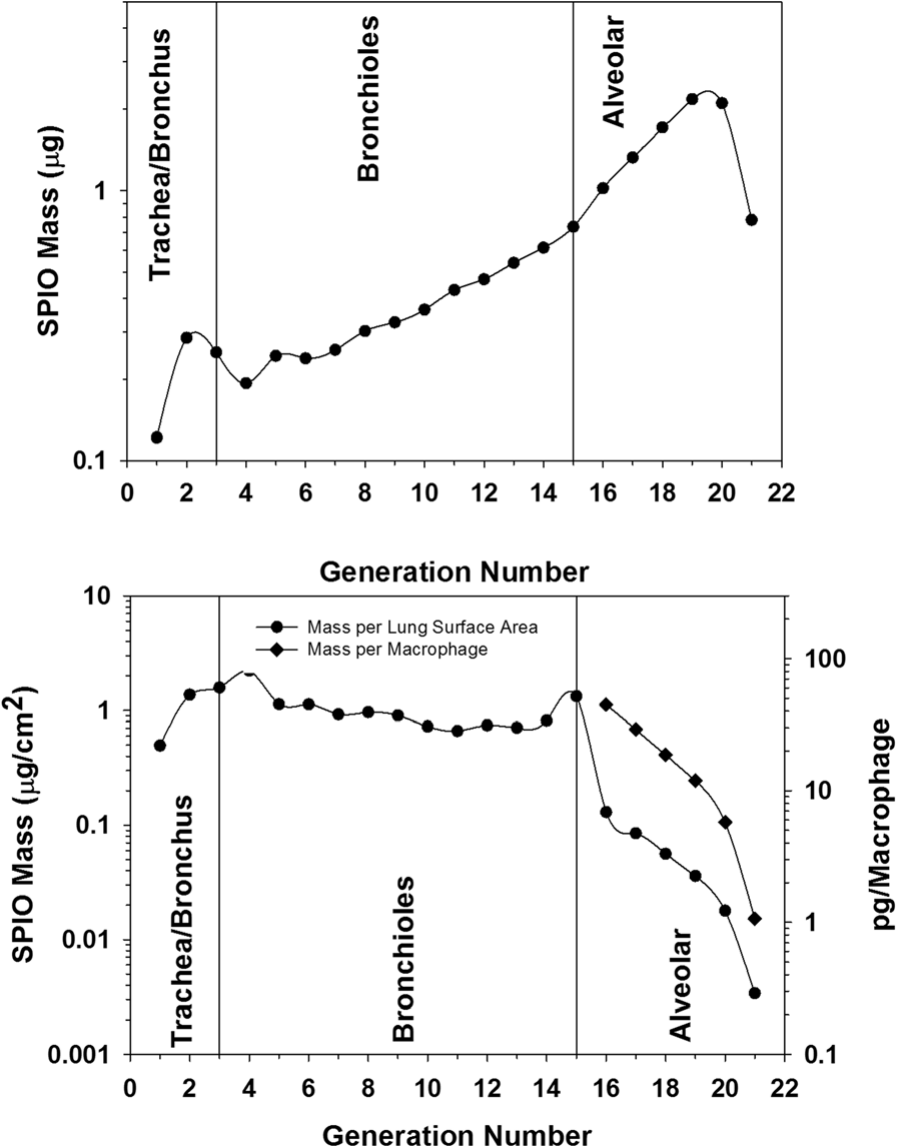
**SPIO nanoparticle mass deposited in each region of the lung (top) and regional dosimetry (bottom panel) calculated with the Balb/c specific version of MPPD.** Target cell doses are presented in units of mass per surface area of cells for all airways, and as pg/macrophage for the alveolar region only to correspond to experimental in vitro dose-response data. Given a density of 5.2 grams/cm^3^, a picogram of SPIO particles would contain 154,015 primary 13 nm diameter particles with a surface area of 8.87 × 10^−7^ cm^2^.

Total inhaled mass calculated from the minute volume, exposure duration, and the gravimetrically measured aerosol mass concentration was 365 μg. Total SPIO nanoparticle mass deposited at the termination of exposure was 14.5 μg (SD 2.6) as measured by magnetic particle detection (Figure 2). The largest fraction of SPIO nanoparticle mass, 62%, was predicted to deposit in the alveolar region (Generations 16-22), followed by the bronchiolar region (34%, generations 3-15), and the trachea/main bronchus (2.8%, generations 1 and 2) (Figure 3). MPPD predicted target tissue doses ranged from 0.003 to 2.2 μg/cm^2^ lung tissue surface area. Target tissue doses were similar throughout the generations comprising the bronchiolar region, near 1 μg/cm^2^ lung tissue surface area (Figure 3). Regional target tissue doses fell considerably in the high surface areas of the alveolar region, from 0.13 in generation 16 to 0.003 μg/cm^2^ lung tissue surface area in generation 22 (Figure 3). Macrophage associated doses calculated by dividing the deposited mass in each alveolar region by the number of present macrophages in the region ranged from 4-100 pg/cell. The influence of assuming 100% uptake by macrophages is addressed in the discussion section.

The net clearance of particles from the lung was monitored over a 168 hour post exposure period (Figure 2). Clearance of particles by degradation, dissolution, or transport out of the lung are all monitored by the magnetic particle detection (MPD) method used to measure tissue mass of SPIO nanoparticles, which is sensitive only to particles, not iron oxide ions. Clearance was linear with a single phase over the 168 hour post exposure period. The absolute clearance rate was 0.05 μg/hour (Figure 2). Normalized to deposited dose, the clearance rate was 8.6% /day.

### Lung response to inhaled SPIO

#### Histopathology

**Figure 4:**
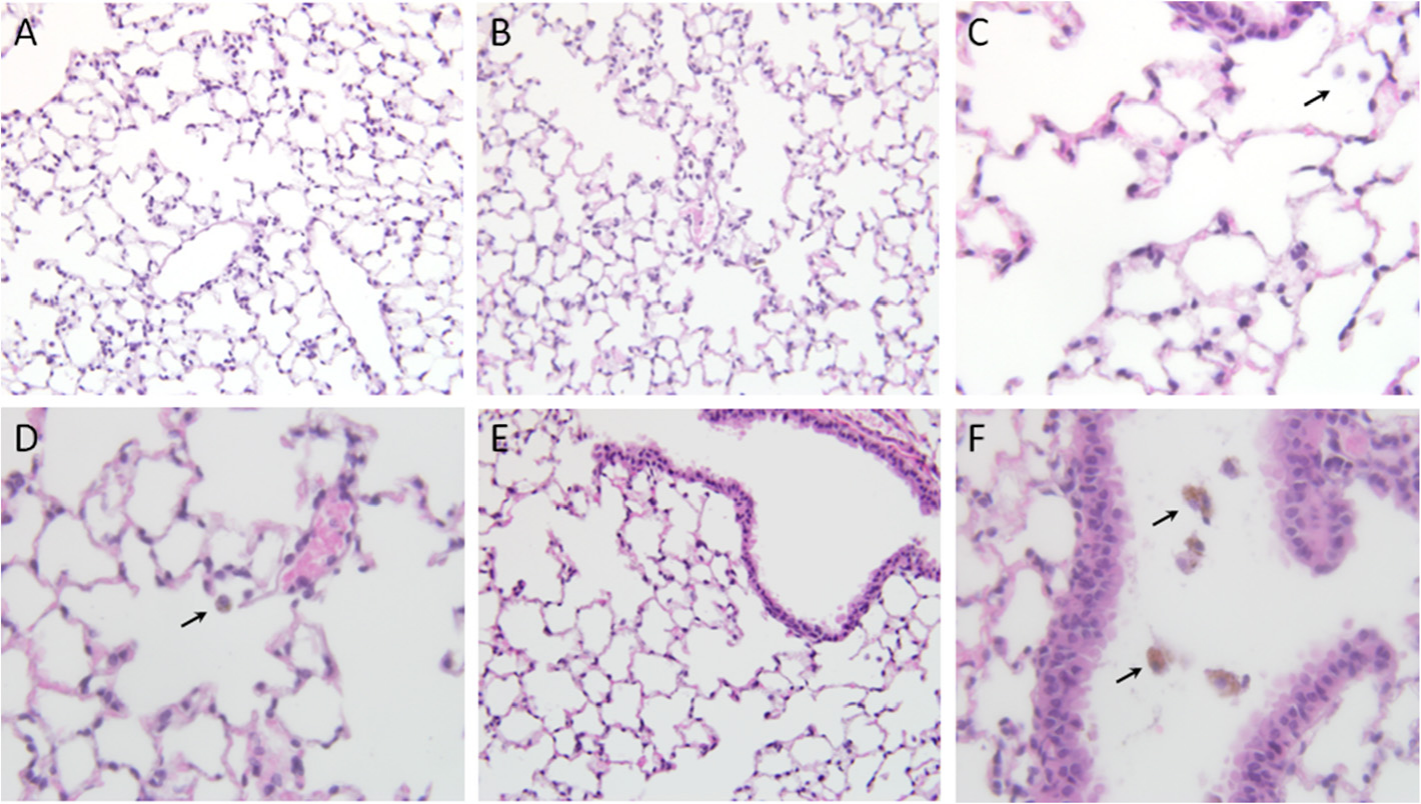
**Representative lung histological sections from control and nanoparticle-exposed mice.** Examples of H&E stained sections obtained from control **(A,C,E)** or nanoparticle-exposed mice **(B,D,F)** at 48 hrs post-inhalation exposure. Arrows highlight the darker brown staining reflecting greatly increased nanoparticle burden observed in alveolar macrophages in treated groups compared to controls. The increase in nanoparticle-laden macrophages observed within the primary bronchus of exposed animals is also highlighted in panel **F**. (Panels **A**, **B**, **E** and **F**: 200×; Panels **C**, **D**: 400×).

Representative micrographs taken from H&E stained histological sections are shown in Figure 4, and results of the histopathology analysis for the individual animals is summarized in Table 1. Overall, the moderate level exposure used in this study resulted in microscopic lesions within the lung characterized by minimal to mild macrophage infiltration and interstitial inflammation. In all nanoparticle exposed animals, increased numbers of diffusely scattered macrophages were observed within alveoli, along with clusters of macrophages near alveolar ducts, terminal bronchioles and within the primary bronchus. Alveolar macrophages were also observed in some animals within the control exposure group. However, the extent of alveolar involvement was greater in exposed animals compared to controls, and macrophages observed in nanoparticle-exposed animals differed significantly in appearance from the control groups, showing dark brown staining of foreign granular material, presumed to be test SPIO nanoparticles. Alveolar macrophage infiltration in exposed animals was most prominent at 48 hour following exposure, and evidence of mild interstitial mixed inflammation was also observed in some exposed mice at the 48 – 168 hour time points.

**Table 1:** **Summary of microscopic lung lesions**

**Sham control**	**Time post exposure (hr)**					
	**0**	**6**	**24**	**48**	**96**	**168**
**Lung, number examined**	**5**	**5**	**5**	**5**	**5**	**5**
Infiltrate Cellular, Macrophage	1^a^ (1.0)^b^	2 (1.5)	3 (1.3)	1 (1.0)	2 (1.0)	5 (1.0)
Foreign Body (intracellular)	0	0	0	0	0	0
Mixed Inflammation, Interstitium	0	0	0	0	0	1 (1.0)
**Nano-particle exposed**						
**Lung, number examined**	**5**	**5**	**5**	**5**	**5**	**4**
Infiltrate Cellular, Macrophage	5 (1.0)	5 (1.2)	5 (1.0)	5 (2.0)	5 (1.0)	4 (1.0)
Foreign Body (intracellular)	5 (1.0)	5 (1.0)	5 (1.0)	5 (1.0)	5 (1.0)	4 (1.0)
Mixed Inflammation, Interstitium	0	0	0	2 (1.0)	2 (1.0)	1 (1.0)

^a^Number diagnosed.

^b^Average severity grade in parenthesis (1 = minimal, 2 = mild).

#### Gene transcription profiling

**Figure 5:**
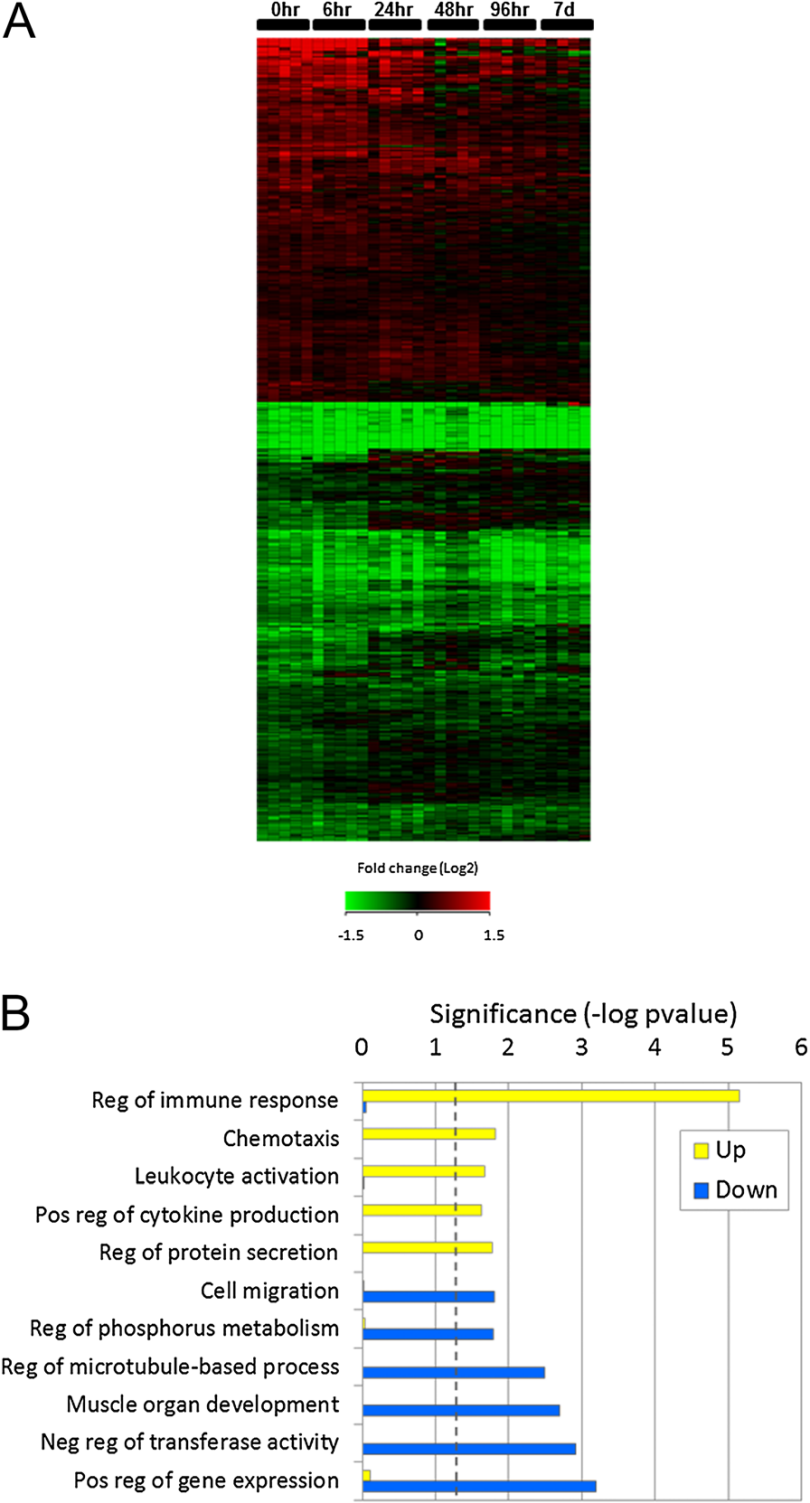
**Transcriptomic analysis of gene regulation by nanoparticles. A)** Hierarchical clustering of significantly effected (compared to time matched controls) transcripts shows a higher preponderance of down regulation (green) by SPIO nanoparticle exposures. Up-regulation (red) was more prominent during earlier time points where the most highly induced mRNAs encoded inflammatory cytokines. **B)** Highest ranking biological processes associated with transcripts up-regulated by in vivo exposure to SPIO nanoparticles. Cellular processes associated with inflammation and clearance of foreign bodies (cytokine production, cell migration, chemotaxis) were significantly upregulated.

**Figure 6:**
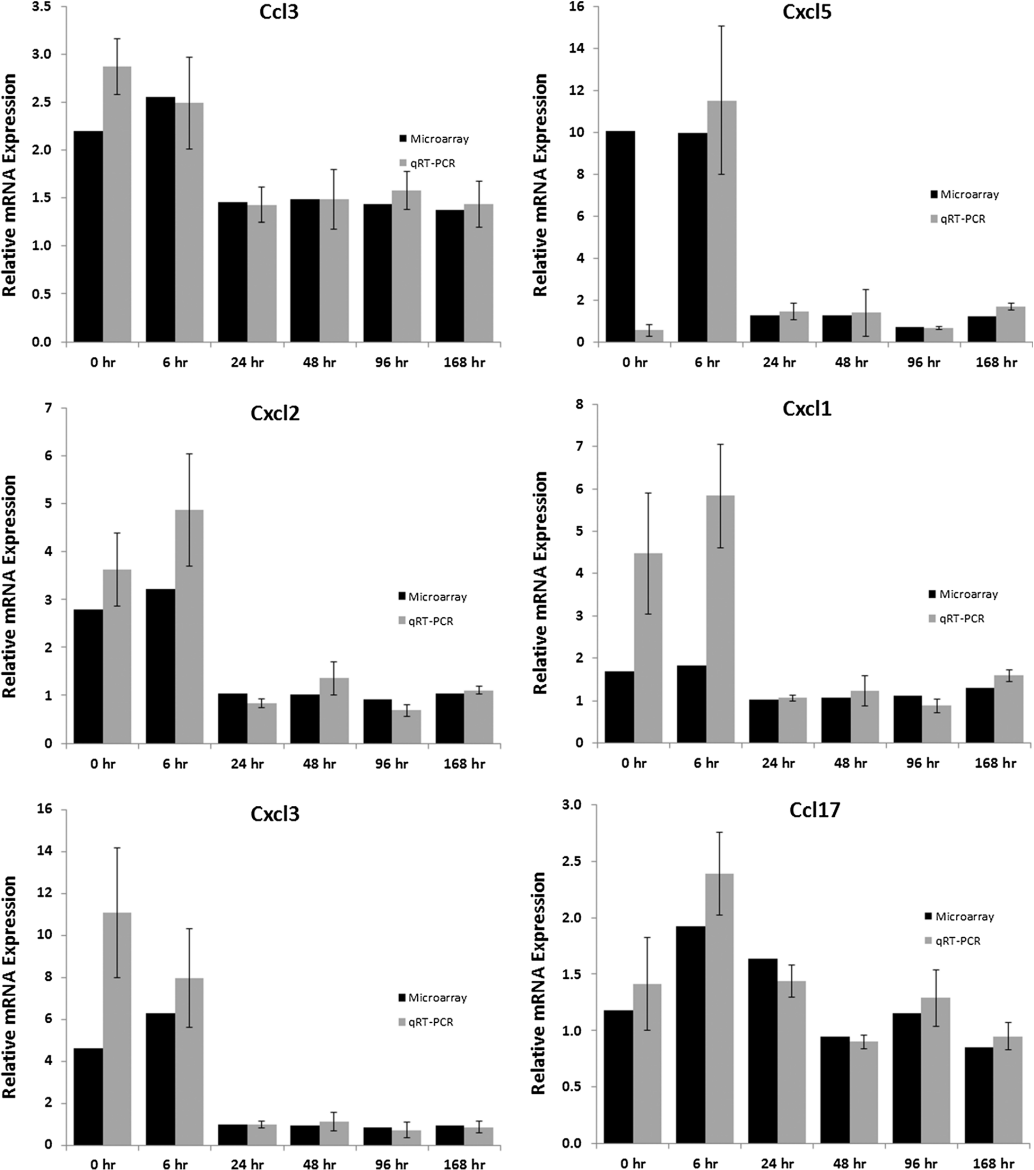
**Microarray data and PCR validation of transient induction of transcripts (compared to time matched controls) for key inflammatory cytokines.** Induction of cytokines was prominent at 4 hours, but resolved by the peak of macrophage infiltration at 48 hours.

In order to identify the molecular impact of the SPIO nanoparticles on the lung tissue, we performed whole genome microarray profiling and biological pathway analysis. We identified 815 genes that were significantly changed in SPIO nanoparticle exposed mice at the end of the 4 hr exposure period (0 hour time point) compared to sham (air exposed) controls (Raw and normalized Affymetrix data files are available online through Gene Expression Omnibus accession GSE51417 (http://www.ncbi.nlm.nih.gov/geo/query/acc.cgi?acc=GSE51417). Hierarchical clustering of these data demonstrates a strong transcriptional response that persists through 6 hour post exposure and gradually declines over the next 7 days (Figure 5A). Evaluation of the “up” and “down” regulated genes reveals that the majority of the up-regulated genes are involved in regulation of the immune response, chemotaxis and leukocyte activation (Figure 5B), consistent with the histopathology results showing infiltration of macrophage cells. Interestingly, the gene expression changes in the primary chemokines CCL3, CXCL5, CXCL1, CXCL1, CXCL3, CCL17, AND CCL9 involved in macrophage recruitment are already resolved to baseline levels by 48 hours (confirmed by QPCR, Figure 6), when the macrophage levels are at their peak, indicating that these chemokines are likely being synthesized by lung epithelial cells as well as infiltrated immune cells. In contrast to the up-regulated genes, many of the down-regulated genes (and associated biological processes) are persistently down-regulated through the 7 days post-exposure, indicating that the SPIO nanoparticle exposed animals are not yet resolved to steady state while they continue to clear the nanomaterials from their tissues.

### In vitro target cell dosimetry

**Figure 7:**
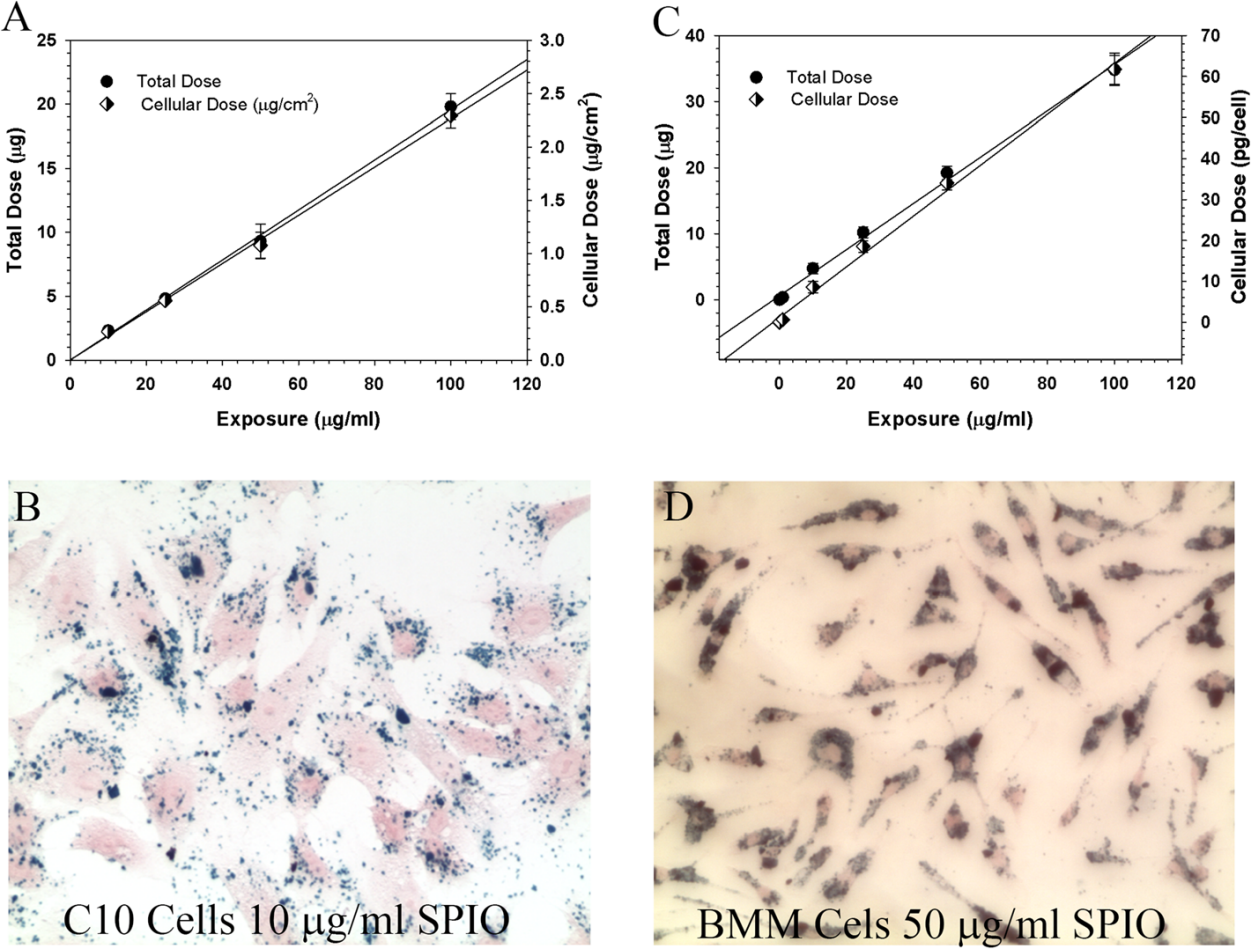
**Cellular SPIO Nanoparticle Dosimetry.** Measured in vitro target cell doses of SPIO nanoparticles in C10 (**A**, Left) and BMM (**C**, right) lung epithelial cells measured by magnetic particle detection. Cell associated SPIO nanoparticles in C10 epithelial cells (**B**, lower left) and BMM (**D**, lower right) after a 4 h exposure. Iron oxide particles are stained with prussian blue and cells are stained with nuclear fast red. Magnification, 20×.

After preparation and characterization SPIO nanoparticles were incubated with C10 lung epithelial cells and bone marrow derived macrophages (BMM) for 4 hrs and uptake was quantified using a Magnetic Particle Detection (MPD) system developed in our lab [11]. A linear relationship between SPIO exposure and cellular dose was observed for both C10 (Figure 7A) and BMM (Figure 7C) cells. Representative images of C10 cells and BMMs treated with 10 or 50 μg/mL of SPIO nanoparticles for 4 hours show the presence of SPIO particles (Figure 7B, 7D). A 24 hour extended time-course of cellular dosimetry was also conducted in C10 epithelial cells to explore the time-dependent and accumulated dose effects on association of SPIO nanoparticles with C10 cells.

### In vitro target cell SPIO delivery and cell association

**Figure 8:**
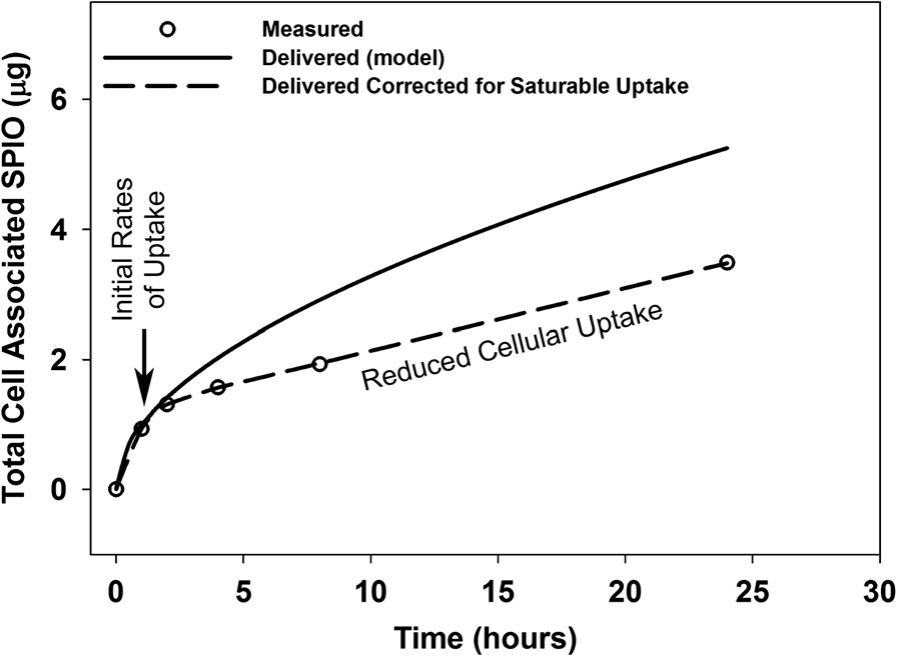
**ISDD simulation of SPIO particle association with C10 epithelial cells in vitro.** Modeled and observed SPIO mass doses corresponded well at early time points, but there was evidence at later time points of the involvement of a dose-related process that reduced SPIO nanoparticle doses relative to the predicted amount delivered. Inclusion of a saturable “uptake factor” led to agreement between modeled and observed doses.

ISDD model simulation of the amount of SPIO nanoparticles delivered to cells over the 24 hour in vitro study was consistent with the measured cell associated material at early time points (1 and 2 hours), but not at later time points (4, 8 and 24 hours) (Figure 8). We interpret the consistency between the initial (early) measured and simulated delivered rates as evidence supporting the accuracy of ISDD for estimating particle delivery during the delivery limited phase of transport. At the later time points, where target cell doses are higher, a reduced fraction of particles predicted to be delivered to cells remained associated with the C10 cells after harvesting. Overall, the fraction reduced from 93% at early time points, to 89% at 4 hours, ending at a value that remained consistent (67-68%) between 8 and 24 hours. Thus, there appeared to be a shift at longer exposures and higher delivered doses towards a reduced rate of uptake, loose association of delivered SPIO nanoparticles with cells or reduction in other process(es) that contribute to the association of SPIO nanoparticles with C10 cells.

### In vitro dose-response for in vivo markers of lung inflammation

**Figure 9:**
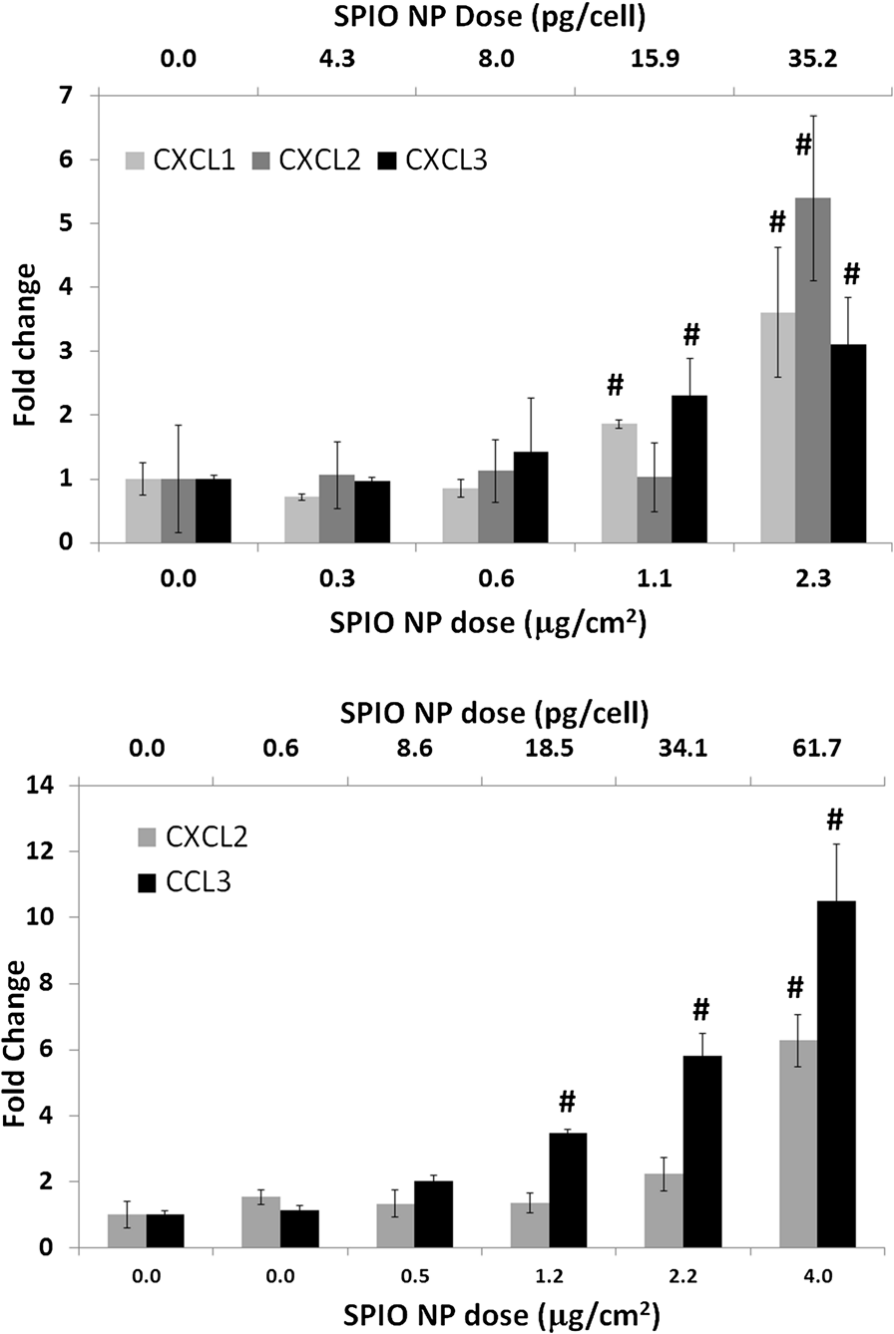
**SPIO nanoparticle induced gene expression in vitro.** Top) A dose dependent increase in gene expression was observed in BMM cells treated with 44-2 carboxy-modified SPIO nanoparticles for 4h. Significance values calculated using Student’s *t-*test. # < 0.01; Bottom) dose dependent increase in gene expression was observed in C10 cells treated with 44-2 carboxy-modified SPIO nanoparticles for 4 h. Significance values calculated using Student’s *t-*test. * < 0.05, # < 0.01.

BMM and C10 cells were treated with SPIO nanoparticles and mRNA expression was quantified using qRT-PCR. There was a dose dependent increase in the expression of CXCL1, CXCL2 and CXCL3 genes (for BMM, Figure 9A) and CXCL2 and CCL3 genes (for C10 cells, Figure 9B). There also appeared to be a threshold dose of ~1.1 μg/cm^2^, below which there was no observable effects on regulation of these transcripts relative to controls.

### Target cell dosimetry-based correlation of in vitro and in vivo responses

We hypothesized that improved relationships between in vitro and in vivo models of experimental toxicology for nanomaterials would result from placing response data in vitro and in vivo on the same dose scale, the amount of material associated with cells (target cell dose). Two regions of mouse lung (bronchiolar, generations 1-16) and alveolar (16-21) were considered and compared to dose-responses in two commonly used, representative cell types, one for epithelial cells (C10, bronchiolar and alveolar regions) and one for macrophages (murine BMM, alveolar region only).

Following four hours of inhalation exposure, target tissue doses of 0.009-0.13 μg SPIO/ cm^2^ led to an inflammatory response in the alveolar region (generation 16-22) characterized by interstitial inflammation and macrophage infiltration. Inflammation was not observed in the bronchiolar region. In vitro, higher target tissue doses of ~1.2-4 μg SPIO/ cm^2^ of cells were required to induce transcriptional regulation of markers of inflammation, CXCL2 CCL3, in C10 lung epithelial cells. The higher doses occurring in the bronchiolar region (1-2 μg SPIO/ cm^2^ of cells), were consistent with those triggering an inflammatory response in vitro in C10 cells, but inflammation was not observed histologically in vivo. Alveolar macrophages contribute to the initiation of inflammation in the alveolar region of the lung [12]. Following SPIO nanoparticle exposures, estimated macrophage SPIO nanoparticle doses ranged from 1-100 pg/cell (Figure 3). Induction of inflammatory markers was observed in vitro in BMM at doses of 8-35 pg/cell, in the middle range of macrophage target cell doses experienced in the alveolar region of exposed mice where macrophage infiltration was observed. Overall, there was a good correspondence between target cell doses triggering inflammatory processes in vitro and in vivo in the alveolar macrophage population, but not in the epithelial cells of the alveolar region.

### Human lung dosimetry: carboxylated SPIO particles would be inflammogenic

**Figure 10:**
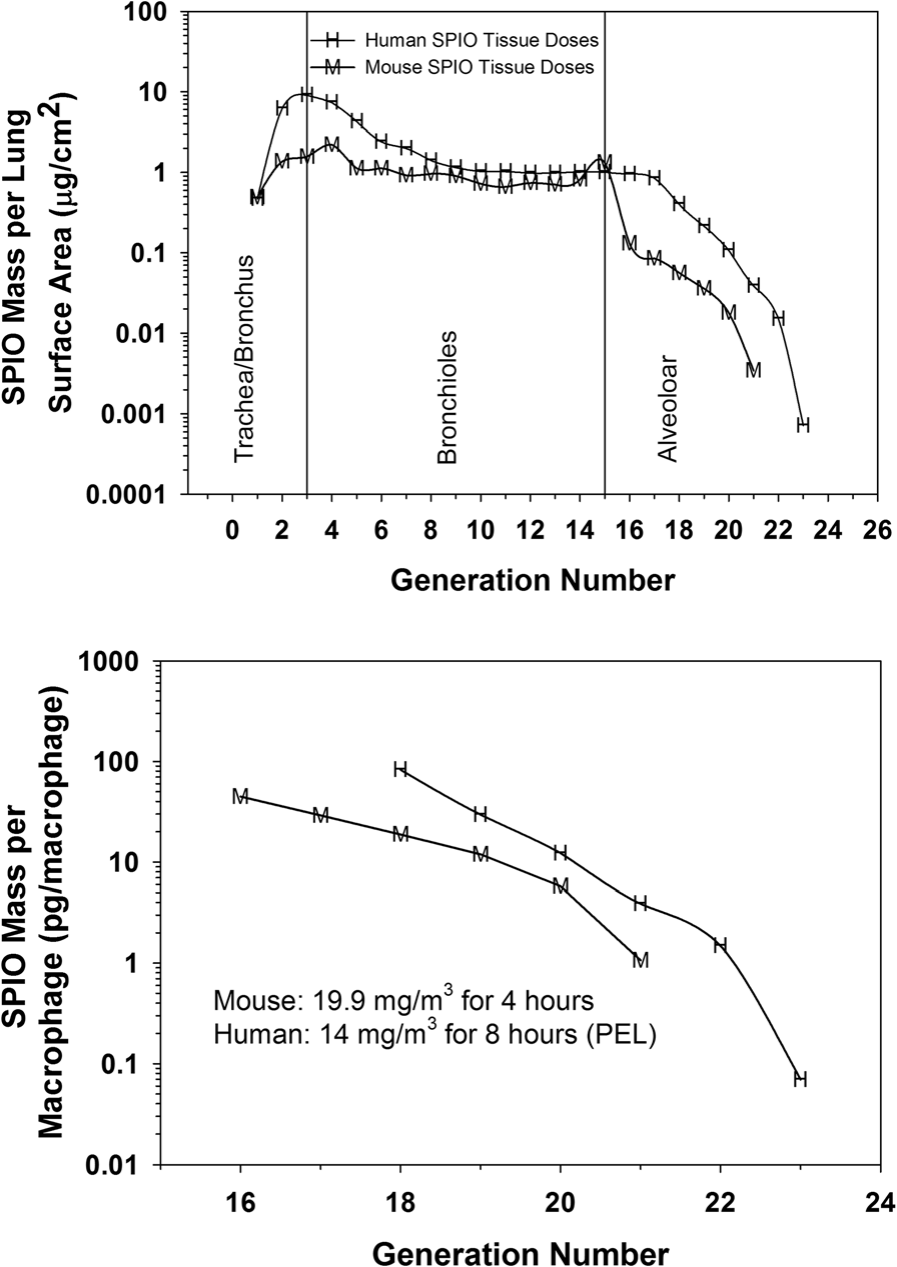
**Comparison of deposited (clearance was not considered) doses per lung surface area and per macrophage from mice in this study to the calculated deposited doses in humans exposed at the PEL.** Regional lung doses on a particle mass per surface area and particle mass per macrophage were similar or higher in the human than the mouse.

We simulated occupational human exposure to the SPIO nanoparticles studied here to allow comparison of regional doses in the human lung with the mildly inflammogentic regional lung doses produced during the 4 hour mouse exposure to 19.9 mg/m^3^ SPIO, and in macrophages and epithelial cells in vitro. Deposited (clearance was not considered) doses per lung surface area and per macrophage were calculated as described for the mouse. The human exposure setting was eight hours exposure to the Occupational Safety and Health Administration (OSHA) Permissible exposure limit (PEL, dust and fume) of 10 mg/m^3^ (as Fe), adjusted for the fractional iron content of the SPIO nanoparticles (0.72) to a final value of 14 mg/m^3^. Regional lung doses on a particle mass per surface area and particle mass per macrophage were similar to or higher than the mouse (Figure 10).

## Discussion

Animal models of toxicity, in particular rodent models, have been the major source of toxicity data used for making quantitative assessments of the risks of pharmaceuticals, particulates, and other environmentally and occupationally important hazards for decades [13]. Intrinsic integration of the processes that lead to toxicity, adsorption, distribution, metabolism, elimination (ADME), and perturbation of physiological, cellular and biochemical systems, confers high relevance of these test systems for assessing the potential for toxicity in exposed humans. Confidence in these systems is highest when comparative data are available regarding metabolism, qualitative and quantitative similarity in key obligatory processes involved in developing the toxicity that show similarity or quantitative differences that can be addressed in the risk assessment process.

With the recent shift towards use of in vitro systems to make specific quantitative assessments of toxicity, or of risk to humans directly [1], understanding the extent to which, or under what conditions in vitro systems adequately recapitulate biology and dose-response of in vivo systems will remain one of the most pressing scientific questions for the field of toxicology for several decades. Cellular responses are the product of interactions of the particles (dose) with specific cellular components and systems (receptors, lysosomes, signaling pathways), where both dose and these biological elements can be or are different between in vitro and in vivo systems. Deconvoluting the influence of these two categories of system differences on biological responses is necessary for both design and interpretation of in vitro particle toxicology studies for prediction of in vivo response.

Conventions and shortcomings of current approaches for measuring and characterizing nanomaterials in situ, particularly in regional measurements in non-homogenous organs such as the lung, are key obstacles to the goal of aligning of in vitro and in vivo exposures on the basis of exposure/dose for comparative nanotoxicology and risk assessment. Inhalation-route exposures are typically characterized and reported as a particulate concentration (mg/m^3^), or total dose/exposure (mg/kg) for intra tracheal or pharyngeal aspiration studies, but units of liquid concentration (mass/ml) are used for reporting in vitro studies of cells representing the respiratory tract [6-8,14]. These representations of exposure, which are conventional and convenient, are not comparable across systems. Qualitatively and quantitatively different physical, physiological and biochemical processes control the time and particle dependent relationship between exposure and target cell dose in in vitro and in vivo systems [15-18].

Utilizing a commercially/medically important iron oxide nanoparticle with unique superparamagnetic properties enabling rapid quantification of cellular dose, we conducted coordinated vivo and in vitro toxicity studies, reporting responses in the whole mouse lung and in two cell types resident in the alveolar region of lung. Biomarkers of the in vivo inflammatory response identified by global transcriptomic analysis of treated mouse lung tissue were assessed in vitro in C10 epithelial cells and bone marrow macrophages.

The iron oxide mass dose available to alveolar macrophages in vivo after 4 hours of exposure to 19.9 mg/m^3^ SPIO nanoparticles ranged from 1 to 50 pg/macrophage, and was sufficient to cause mild, transient inflammation. The increased expression of mRNA for the chemokines CXCL2 and CCL3 observed in lung tissue from BALB/C mice at these doses was also observed in vitro in mouse macrophages at target cell doses of 18.5-61.7 pg/macrophage. We cautiously conclude that applying contemporary concepts of target tissue dosimetry reveals an apparent consistency in the cellular dose of SPIO particles required to initiate inflammatory responses in vivo in the whole lung and in vitro in macrophages. As key modulators of the inflammatory response in the lung, it is plausible that induced inflammatory signaling of the type we observed in vitro by macrophages would initiate the pulmonary inflammation we observed in vivo in mice exposed to the same particles. However, this study was designed to look for correlations between whole-lung inflammatory response and response of macrophages in vitro, which we observed, and cannot draw conclusions about the specific role any lung cell type played in the evolution and resolution of the inflammatory response. Furthermore, because we observed consists inflammatory responses in vitro and in vivo, and we considered one particle type, we did not consider the potential inhibitory effect of soluble iron oxide on inflammation [19]. In this assessment, we compare the SPIO nanoparticle dose available (delivered to the alveolar region where the macrophages reside) to macrophages in vivo to the measured SPIO nanoparticle macrophage dose measured in vitro. Light microscopy revealed that in vitro and in vivo, macrophages contained visible amounts of SPIO nanoparticles comprising a significant amount of their intracellular volume. While this observation supports the comparability of the in vitro and in vivo doses, others have shown that alveolar macrophages scavenge a fraction of particles in the alveolar region ranging from 20-80 percent, with a strong size dependence [20]. The fraction of particles the size (mass median diameter, SMPS) of SPIO nanoparticle agglomerates inhaled in our experiment, 171 nm, falls between the two closest values reported by Oberdorster et al. [20], 80 nm and 500 nm, for which fractional macrophage scavenging is reported. Assuming a fractional scavenging between these two values of about 50% would reduce our computed macrophage doses to 9-31 pg/macrophage, still within the range of macrophage doses causing inflammation in vitro. The low number of lavageable alveolar macrophages in the mouse (~10^5^) [21], tissue requirements for pathology, transcriptomic analysis, and particle clearance studies, precluded direct measurement of SPIO nanoparticles in lavaged alveolar macrophages by MPD at the time of the study design. The alveolar macrophage content of larger microscale particles has been measured by light microscopy in the macrophages of total lavaged cells in humans [22,23] an approach that was not suitable for our nanoscale SPIO particles.

Measured SPIO mass cellular doses stimulating statistically significant increases in transcripts for CXCL1, CXCL2 and CXCL3 in epithelial (C10) cells, in vitro, were one to three orders of magnitude higher than inflammogentic doses in vivo in the pulmonary epithelium (1.1-2.3 μg/cm^2^ vs. 0.003-0.1 μg/cm^2^) which altered expression of the same biomarkers. Locally higher doses in vivo occur at airway bifurcations in the upper airways. Balàshàzy used CFD modeling of particle deposition in human airway generations 3-4 to compute the enhancement factor, the ratio of local dose to the average surface area dose in the region [24]. The enhancement factor is highly dependent on the size of the area considered, ranging from ~ 5-65 for 200 nm diameter particle as the deposition area decreases from 100 μm to 3 μm. Adjusting the regional average doses computed by MPPD in the upper airways by the enhancement factor for 200 nm diameter particles for an area 10 × 10 cells (100 μm), which is 65 [24], we would calculate mass SPIO doses of 6.5 μg/cm^2^ in small areas of the lung. These target cell doses align more closely with those upregulating expression of the chemokines CXCL1, 2 and 3 in C10 cells in vitro. However, the small area of cells experiencing these target cell doses may or may not be a large enough fraction of the total lung to be considered a plausible contributor to the inflammatory response we observed in vivo. Indeed, the upper airways themselves (generations 1-4 in Balàshàzy’s analysis) are a very small fraction of the total mouse lung (0.2%); the hot spots represent an even smaller fraction of the surface area of the upper airways.

In contrast to SPIO nanoparticle cellular doses in vitro, which were directly measured by MPD, cellular doses were computed from the total deposited doses measured using MPD by applying established tools for calculating regional lung deposition of particles. The potential for differences between our computed in vivo cellular doses and actual doses could be evaluated or overcome if experimental methods for measuring cellular doses of particles in the lung were available. Tools and approaches for measuring regional lung doses currently are limited by the ability to resolve dose on a regional basis. Magnetic resonance imaging applied to SPIO nanoparticles has been used to measure regional dosimetry in rats and mice, but only with resolution sufficient to separate central from peripheral lung, not by airway generation [25,26]. Sectioning methods [27], requiring serial sectioning of the lung and semiquantative evaluation of deposited dose by microscopy were beyond the scope of this effort and have their own inherent limitations [27].

The current 10 mg/m^3^ (as Fe, 14 mg/m^3^ as Fe_2_O_3_) OSHA PEL for Iron (II) oxide (iron oxide fume, iron (III) oxide, ferrous oxide) is based on a finding of no effects on alveolar epithelial permeability, a marker of pulmonary inflammation and/or injury, in humans exposed to an aerosol of iron oxide particles (MMAD, 1.5 μm, estimated CMD of 179 nm using an agglomerate density of 2.5 (John Lay, personal communication) and soluble iron [28]. The particles were agglomerates of colloidal iron oxide particles (size not reported), suspended in liquid and nebulized (liquid drop size, ~ 5 μm MMAD, John Lay, personal communication) for the human exposures. Inconsistent (increases and decreases), but not statistically significant changes in epithelial permeability were reported in volunteers exposed by mouth breathing to an average of 12.7 mg/m^3^ (range 9-16 mg/m^3^, determined gravimetrically) for 30 minutes [29]. The suitability of PEL’s and similar exposure standards derived from studies of microscale materials for use in regulating exposure to nanoscale materials is an important issue that has been explored in depth only for TiO2 [30]. Comparing calculated regional lung and macrophage doses of nanoscale SPIO particles causing effects in our murine in vitro and in vivo model systems with those calculated for the human exposures to the same material at exposures equal to the current PEL for iron oxide (Figure 10), revealed that based on mass doses alone, transient inflammation might be anticipated in humans. Extending this analysis, we also compared estimated (simulated using MPPD) mass iron oxide particle regional lung tissue doses resulting from the exposures to the somewhat larger iron oxide particles used to derive the PEL [29] to those that would result from exposures to the aerosol used to expose mice in our study. Regional deposited mass doses predicted by MPPD were very similar for exposures to these two particles (Additional file 7) for a 30 minute exposure period, assuming a GSD of 2.1 for the nebulized particle in the Lay et al study. This would appear to support the conclusion that exposures to the nanoscale material would not be expected to be of greater risk than exposure to the microscale iron oxide agglomerates if mass dose alone were the determinant of biological response. The similarity in the deposited mass doses alone should not be considered sufficient evidence to conclude that exposure standards for nanoscale materials can be derived directly from studies using microscale materials. The intrinsic biological potency of the nano- and microscale iron oxide particles could be different and of course the doses on a surface area basis may also be significantly different. For example, a comparison of delivered particle surface area might reveal that the nanoscale iron oxide particle exposures lead to much higher deposited surface area than would be expected in the microscale particle exposures. The absence of information on the size and thus the number and surface area of the primary particles forming the microscale agglomerates precluded our calculation of total number and surface area of particles deposited in the Lay et al study. We elected not to approximate surface area of the formed agglomerates in the aerosol because the resulting value would underestimate the total surface area of the deposited particles.

The availability of experimental (e.g. ICPMS, MPD) and computational (e.g. ISDD, MPPD) methods for measuring or calculating target cellular doses of micro and nanoscale materials for rodent in vivo and in vitro systems now enables comparison of cellular/tissue responses between these systems on the basis of common measures of dose, e.g. mass, surface area or particle number delivered to cells. With minimal information on particle and test system characteristics, ISDD and MPPD can also be utilized to optimize the design of coordinated in vitro and in vivo studies for comparing responses and testing in vitro systems for their power to predict in vivo responses.

## Conclusions

With the recent extension of MPPD to the mouse [31], it is now possible to use MPPD and ISDD or experimentally measured doses, as we have done here, to compare responses between in vitro and in vivo rodent toxicity test systems cell on the basis of particle dose to one or more common target cells. We find that the macrophage cellular doses of 12.8 nm (68.6 nm CMD in aerosol form) SPIO causing inflammation and increased expression of CXCL2 and CCL3 in vivo corresponded well to cellular doses increasing expression of the same markers of inflammation in murine macrophages in vitro. In contrast, even considering potentially higher doses at airway bifurcations, doses required to cause increased expression of inflammatory markers in C10 epithelial cells in vitro were much higher than those calculated in the lungs of mice exposed by inhalation to the SPIO particles. The similarity between the mildly inflammogentic cellular doses experienced by mice in this study and those expected in humans exposed to the same SPIO particles by inhalation at the existing PEL of 10 mg/m^3^ iron oxide (as Fe) for 8 hours imply a small margin of exposure ([exposure causing effects]/[actual exposure]) and for pulmonary inflammation in humans for these particles. Improving the approach for the respiratory tract presented here by further refining the measures of cellular dose, increasing the specificity of cellular response, and extending to additional particles would be valuable next steps toward elucidating the relationships between in vivo and in vitro responses.

The approach described here is generally applicable, with adaptations, e.g. tissue selection, endpoint selection, to other nanomaterials, or target tissues. The general computational dosimetry framework for relating doses across particles an systems address particle differences in density and size, as well as the geometry of the system (respiratory tract, cell culture dish), and so are also widely applicable with some adaptations.

## Materials and methods

### Study overview

The research program was anchored in a short-term inhalation study conducted in Balb/c mice. Inhalation was selected rather than IT or oropharyngeal (OP) so the effects of particle size and lung deposition could be accounted for in the context of physiologically normal exposures. All exposure and mouse respiratory characteristics were measured and total inhaled dose was measured to allow calculation of lung target tissue doses. Respiratory tract particle deposition modeling was applied to calculate lung region specific doses for comparison to in vitro dose-response studies. Global transcriptomics was conducted in parallel with histopathology to identify PCR targets for the most robust in vivo responses (pathology) that could then be applied to the in vitro dose-response system. Two mouse derived cell types, one representing lung epithelium (cell line) and one representing lung macrophages (primary macrophages), were used in dose-response studies covering the range of per cell or per cell surface area nanoparticle doses calculated for the in vivo study. Transcriptional regulation of cytokines identified in the in vivo study was used as the response metrics in the in vitro studies to assure similar responses were being compared across systems. Dose-responses in regions of the mouse lung containing the cell types used in the in vitro studies were compared to the in in vitro responses to determine if in vivo and in vitro responses corresponded when tissue dosimetry was applied.

### Particle synthesis and characterization

Superparamagnetic iron oxide (SPIO) nanoparticles (Fe_3_O_4_) were prepared by chemical co-precipitation [32] at 23°C [32]. Briefly, 0.2 M FeCl_3_ (anhydrate, EM Science) and 0.1 M FeSO_4_∙7H_2_O (J. T. Baker) were mixed in 100 mL H_2_O under N_2_. Then 10 mL of 29.5 wt% NH_3_.H_2_O (J. T. Baker) was injected into the mixture with vigorous stirring. Upon addition of NH_3_.H_2_O, an immediate brown to black color change implied formation of iron hydroxide in the solution. After the solution was dehydrated (overnight stirring) SPIO colloidal nanoparticles were collected by precipitation with a 2” × 2” × ½” neodymium magnet (Magnetics, Inc.) and washed three times with deionized H_2_O.

To enhance surface charge density, the SPIO nanoparticles were surface-modified by covalent binding of –COOH groups through. The washed Fe_3_O_4_ nanoparticles were dispersed in a 250 mL mixture of ethanol and H_2_O (volume ratio 20:1) pH adjusted by addition of NH_3_.H_2_O, to pH ~ 8.5 followed by the dropwise addition of 2 mL of silane, N(trimethoxysilylpropyl)ethylenediamine-triacetic acid-trisodium salt (35% in water, Gelest), with stirring. After 12 hours, the reaction mixture was adjusted to pH ~3.5 by addition of HCl and the reaction was allowed to continue to completion (12 hours). The functionalized Fe_3_O_4_ nanoparticles were collected by precipitation with a magnet, resuspended by sonication and washed with deionized H_2_O. To remove ionic and magnetic impurities, this process was repeated until the solution reached pH ~7. Deionized H_2_O (Barnstead nanopure water, resistance >18MΩ∙cm) was used throughout the experiments, and all reactions were occurred under N_2_ atmosphere or in closed containers to protect samples from oxidation.

Synthesized SPIO nanoparticles were imaged by TEM (Zeiss, Libra 120) and SEM (Zeiss Merlin), and the size and zeta potential in water was determined by dynamic light scattering (Brookhaven ZetaPlus analyzer).

Methods used to characterize particles in the aerosol and liquid experimental systems are presented in the corresponding in vivo and in vitro sections, below.

### Cell and tissue SPIO nanoparticle measurement

The cellular content of SPIO nanoparticles in cells (cell culture) and Balb/c lung tissue and feces was measured using a custom-built magnetic particle detector as described previously [11]. The detection system measures iron oxide associated with particles, but not dissolved iron species, which lack the superparamagnetic property required for detection. Serial dilutions of stock nanoparticle suspensions were used to generate a standard curve for SPIO nanoparticle content Standard curves were made in the appropriate biological matrix, for cell culture unexposed cells (Additional file 8A), and for mouse tissues (Additional file 8B). For in vitro studies, the measured cellular content of SPIO nanoparticles was normalized to the number of cells and the surface area of the dish for comparison to approximations of deposited doses in the respiratory tract of Balb/c mice. The total cellular content of SPIO particles was measured directly in one lobe of the lung and scaled by weight to total lung content (the trachea was excluded from analysis of deposited SPIO particles).

### Mouse inhalation exposure

#### General study design

Male Balb/c mice were exposed to aerosolized SPIO nanoparticles using a nano-aerosol generation and inhalation system developed and characterized as described below. Exposure was performed using two groups of 30 male Balb/c mice (60 total). One group of 30 was exposed to aerosolized SPIO nanoparticles and the other served as a sham control, exposed only to the experimental atmosphere [helium-oxygen-humidified air mixture]. Groups of five mice were sacrificed at time 0, 6 hours, 24, 48, 96 hours, and 7 days after exposure. All animal treatments followed protocols approved by The Pacific Northwest National Laboratory institutional animal care and use committee.

#### Aerosol generation and characterization

The SPIO nanoparticle aerosol was generated from the prepared water suspension of carboxylated SPIO (~12.8 nm core diameter) nanoparticles using a custom designed generator (Additional file 9). Pressurized helium (~80 psi) was utilized as a carrier-gas for a single-jet Collision type nebulizer (BGI Inc., Waltham, MA). The SPIO nanoparticle containing aerosol was directed to a specially designed plenum where it was dried and diluted with HEPA filtered air and oxygen. After passing through the mixing-drying plenum, the SPIO nanoparticle aerosol was delivered to the main delivery line supplied with several sampling/monitoring ports. The main delivery line was connected to a 4-tier nose-port exposure carousel (8 nose-ports per tier, 32 total ports for animal exposure and monitoring of exposure parameters (Additional file 9). Each nose-port received ~0.5 L/min for a total of breathing atmosphere therefore processing ~16 L/min through the 32 port carousel.

Aerosol concentration and particle size were monitored in real-time using a Scanning Mobility Particle Sizer (SMPS, TSI Inc, St Paul, MN, US). Spatial uniformity of the nano-aerosol distribution across the nose-only exposure unit was confirmed using SMPS measurements taken at the different nose-ports within and between tiers (Additional file 9). Aerosol was generated for the duration of the 4 hour animal exposure. The temporal stability of the SPIO nanoparticle aerosol exposures was assessed by gravimetric filter analysis, SPMS (CMD, MMD) and Nano Micro Orifice Uniform Deposition Impactor (MMD only, MOUDI (Model 125 B Nano-MOUDI-II, 10 LPM, size range from 10 nm to 10 microns, MSP Corp., Shoreview, MN).

Filter samples were taken each hour of aerosol generation at each of two filter sampling ports at the nose-only terminus. MOUDI data analysis was conducted using house-developed MS-Excel based software that accounts for the effective cut-off diameters (ECD) shift due to the helium-oxygen-air mixture properties. Samples collected on MOUDI stages were also sent for Scanning Electron Microscopy (SEM, Zeiss Merlin) analysis. Temperature and relative humidity of the exposure atmosphere were monitored throughout the exposure period.

#### Acclimation

Mice were housed in solid bottom cages upon arrival to the facility for 1 week prior to the study. Mice were acclimated to the nose only restraint tubes on the two consecutive days (15 minutes, and 30 min respectively) prior to the SPIO nanoparticle exposures.

#### Inhalation exposure

Mice were acclimated to the exposure system for the two days prior to the SPIO exposures. On each day, mice were placed in nose only restraint tubes that fit onto the exposure carousel. Once in the carousel, mice were exposed to a humidified mixture of helium, oxygen, and HEPA-filtered air for ~1 hour on day 1, and 2 hours on Day 2. The following day, mice were exposed to the SPIO nanoparticle aerosol, or a sham control (i.e. just humidified helium-oxygen-HEPA-filtered air atmosphere), for 4 hrs. Atmospheric gas composition for all exposures utilized a helium, oxygen, air mixture with volume fractions of approximately 0.5, 0.125, and 0.375 respectively. Generally, this provided ~20% oxygen (by volume).

##### Respiratory physiology

Prior to inhalation exposure, five mice per treatment group were selected from rodents scheduled for sacrifice after 4 days (see *Sacrifice* schedule below). Each mouse was placed in a plethysmograph that fits on the carousel and base-line respiratory physiology parameters (tidal volume - TV, respiratory rate - RR, minute volume - MV) was measured for 10-min prior to starting exposure. The same mice were monitored for the first 30 minutes of exposure. After 30 minutes of measurements during exposure, mice were placed back into the standard nose-only restraint tubes and exposure was continued. Just prior to the final 30-mins of the 4 hr exposure, the same 5 mice per treatment group were returned to the plethysmograph tubes for final physiology measurements while exposure continued. During the transfer, time off exposure was minimal (less than 1 minute). The data were derived from respiratory flow measurements using whole-body plethysmography [33] and the Buxco Biosystem XA® pulmonary physiology software (Buxco Electronics Inc., Wilmington, NC). Minute volume and the actual exposure concentration were used to estimate total inhaled mass (TIM).

##### Sacrifice

All mice were sacrificed with an overdose of sodium pentobarbital at their assigned post exposure time point, (0 hour, 6 hour, 24 hour, 48 hours, 96 hours, and 168 hours (7 days).

##### Metabolism cages

Mice assigned to the Day seven sacrifice were placed into metabolism cages following SPIO nanoparticle exposure for urine and feces collection. They remained in the metabolism cages for the duration of the study. Feces and urine were collected daily, weighed, and stored frozen at ~-70°C for subsequent analysis. While in the metabolism cages, mice were provided with a certified liquid diet (BIOSERV, AIN-76) rather than pelleted rodent chow to prevent excessive contamination of samples from pelleted feed. Fresh liquid diet was given daily. These mice were acclimated to the liquid diet for ~ three days prior to their placement into the metabolism cages. Testing conducted prior to study indicated that mice readily consumed the liquid diet and maintained a normal weight while on the diet.

#### Histopathology

Following sacrifice by sodium pentobarbital overdose, the lungs were removed, weighed, and the right diaphragmatic lobe was quickly stored in RNAlater for subsequent microarray analysis. The apical lobe was tied off from the tracheal tree and the remaining lobes were weighed and stored frozen for MPD analysis. The apical lobe was inflated with 10% neutral buffered formalin and stored for histopathology analysis. Microscopic evaluation was performed on hematoxylin and eosin (H&E)-stained apical lung lobe tissue sections from five animals from each post-exposure time point sham control group and SPIO nanoparticle exposure group. The severity of macrophage infiltration of the lung was graded based on the extent of involvement of the alveoli, with <10%, or 10-30%, of the alveoli involved containing one or two macrophages representing minimal, or mild, respectively. The severity of inflammation of the lung was also graded based on the extent of involvement of the interstitium, with <10%, or 10-40% of the lung involved, representing minimal, or mild inflammation, respectively.

#### Regional dosimetry modeling

An extension of the Multipath Particle Deposition model (MPPD) for the Balb/c mouse [31] was used in conjunction with measured particle and aerosol characteristics, exposure duration and mouse body weight to calculate the amount of SPIO nanoparticles deposited per surface area of cells in each lung generation. The following parameters were used to simulate the deposited dose: Breathing rate, 317 breaths/minute, tidal volume, 0.24 ml/breath, particle diameter, 68.6 nm (CMD, GSD =1.65), particle density, 5.2 g/cm^3^, concentration 19.9 mg/m^3^, exposure duration 4 hours. The fraction of total SPIO nanoparticles deposited in each lung generation was derived from MPPD output by dividing the predicted deposited mass in each generation by the total mass of SPIO nanoparticles deposited in the lung. The MPPD derived fractional deposition was multiplied by the experimentally measured total deposited mass to determine the regional deposition of SPIO particles. The surface area normalized deposited dose as calculated by dividing the deposited mass in each region by the surface area of the corresponding region. Regional surface areas are provided as supplemental data (Additional file 10). The macrophage dose was calculated by dividing the total deposited dose in each region by the number of macrophages in that region. The total number of alveolar macrophages was 1.25 × 10^6^. Macrophages were distributed in proportion to the number of alveoli in each region. The guiding assumption being that in general, macrophages effectively scavenge the majority of deposited particles in the deep lung, though the efficiency can vary significantly by particle size [20]. The influence of this assumption is addressed in the discussion. Properties of each lung region (e.g. surface area, volume, number of alveoli and the number of macrophages are available as output from the MPPD model [31].

#### Transcriptional profiling

The right diaphragmatic lobe was from 5 mice per group was removed and quickly stored in RNAlater for microarray analysis. Total RNA was extracted from lung tissue (preserved in RNAlater, Invitrogen, Grand Island, NY) using the Qiagen RNeasy Mini kit (Qiagen, Valencia, CA). RNA quality was verified using an Agilent 2100 Bioanalyzer (Agilent Technologies, Santa Clara CA). Biotin-labeled cRNA was synthesized and fragmented using Affymetrix 3′ IVT Express reagents for hybridization to Mouse Genome 430 2.0 GeneChips (Affymetrix, Santa Clara, CA). After hybridization, the arrays were washed and stained with streptavidin-phycoerythrin, and then scanned at a resolution of 2.5 μm using an Affymetrix GeneChip Scanner 3000. Quality control parameters were assessed throughout the experimental process to measure the efficiency of transcription, integrity of hybridization, and consistency of qualitative calls. The synthesis and fragmentation of cRNA were assessed using the Agilent 2100 Bioanalyzer. Spike-in control transcripts were monitored to verify hybridization integrity.

The raw data files were normalized using the Robust Multi-Array Analysis [34], and probesets corresponding to significantly regulated genes were identified using GeneSpringGX software (Agilent Technologies, Santa Clara CA) which incorporates multiple testing and Benjamini Hochberg false discovery rate (FDR) correction, as we have done previously [35]. Statistical significance was determined by ANOVA with unequal variance and Tukey’s HSD *post hoc* test at *p* < 0.05 for SPIO exposed groups compared to respective time-matched sham. Hierarchical cluster analysis of microarray data was performed using Multi-Experiment Viewer [36] software based on log2 expression ratio values. Gene set enrichment statistics for “up” and “down” regulated genes were determined using the DAVID bioinformatics web portal [37] with functional annotation clustering at high stringency. Unless otherwise stated, only biological process clusters that passed a p-value of <0.05 and represented at least 5 genes were considered significant. Raw and normalized Affymetrix data files are available online through Gene Expression Omnibus (http://www.ncbi.nlm.nih.gov/geo/).

#### Real time quantitative RT-PCR

Expression of individual mRNAs was measured by real-time quantitative RT-PCR (qRT-PCR). Complementary DNA was synthesized from total RNA via reverse transcription using the Quantitect kit (Qiagen, Valencia, CA), which includes reagents for genomic DNA removal. qRT-PCR validation was performed on the same RNA samples used for transcriptomic analysis. To further ensure the specificity of the amplifications, primer pairs (Additional file 11) were designed to span introns except for the genes that had intron-exon structures that did not allow this. PCR reactions were carried out using Power SYBR Green Master Mix reagents according to the manufacturer's instructions (Applied Biosystems, Foster City, CA) in an Applied Biosystems StepOnePlus cycler. Cycle parameters were: enzyme activation for 10 min at 95°C, followed by 45 cycles of denaturation at 95°C for 15 sec and annealing/extension at 60°C for 60 sec. Melting curve analyses were performed in each run. Relative expression was determined using the ΔΔC_T_ method with samples normalized to the expression level of the cyclophilin A transcript, the product of the mouse Ppia gene. All analyses were performed on n = 5 samples.

### Cell culture

#### Particle characterization

A 50 μg/ml stock solution of SPIO nanoparticles in cell culture media was prepared for use in both characterization and cell culture studies. Briefly, 100 μl of a 50 mg/ml stock solution in water was placed in a 50 ml centrifuge tube to which 2 ml of fetal bovine serum was added, and the mixture was vortexed for 20 sec. The final 50 μg/ml stock solution was produced by addition of 17.9 ml of phenol red free RPMI supplemented with 2 mM L-glutamine and Pen-Strep (100 U/ml penicillin, and 0.1 mg/ml streptomycin) followed by brief vortexing (20 sec). Particle size and zeta potential were measured using the ZetaPALS Particle Size Analyzer (Brookhaven Instruments Corp., NY). Particle size and Zeta potential values represent the average of at least five separate runs.

#### Cell isolation and preparation

Stock cell cultures of the C10 mouse alveolar epithelial line were maintained in RPMI medium supplemented with 10% fetal bovine serum (FBS), 2 mM L-glutamine, 100 U/ml penicillin, and 0.1 mg/ml streptomycin in a humidified atmosphere of 5% CO_2_-95% air at 37°C.

Primary bone marrow macrophages (BMM) were obtained from bone marrow cells isolated from male C57/BL mice following CO_2_ asphyxiation. Bone marrow cells were flushed from isolated femurs using 5 ml of RPMI 1640 (Invitrogen 11835) + 2 mM L-glutamine (Invitrogen 25030149) + 100U/ml Pen-Strep (Invitrogen 10378016) + 10% fetal bovine serum (Atlanta Biological) using a 25-gauge needle into a 50 mL centrifuge tube on ice. Bone marrow cells were centrifuged, and cultured at 6 × 10^6^ cells on 100 cm^2^ dishes with 10 mL RPMI 1640 supplemented with L-glutamine, Pen-Strep, 10% FBS and 20% conditioned media (see below). Every two days, cells were washed with PBS to remove non-adherent cells and fresh media was added to the dishes. Seven days after isolation, adherent cells representing the BMM enriched population were ready for experimental use.

Conditioned media was used to promote BMM differentiation [38]. L929 fibroblasts were grown to confluence in RPMI 1640 + 10% FBS for seven days after which the supernatant was collected, centrifuged to remove cellular debris, filtered (0.2 μM) and retained. Fresh media was added to the cells and the supernatant collection was repeated 7 days later.. Conditioned media pooled from the collections was kept at -20°C until used in the BMM cell differentiation protocol.

#### Exposure-response

For nanoparticle treatment experiments cells were seeded in 6-well tissue culture treated plates at 1.6 × 10^6^ cells/well and 2.5 × 10^5^ cells/well for BMM and C10 cells respectively, and allowed to attach overnight. Cells were seeded to achieve ~90% confluency at the start of nanoparticle treatment. All treatments were conducted in triplicate.

Immediately before treatments nanoparticle dosing solutions were prepared by serial dilution and brief vortexing of a 50 μg/ml stock solution (see Characterization, above). Dilutions were made in the cell culture media. Old media was removed from cell culture plates and 2.5 mL of nanoparticle solution was added to each well and cells were incubated at 37°C for the duration of the experiment. After four hours of exposure cells were washed 3× with PBS, harvested using 0.25% trypsin, centrifuged and resuspended in 100 μL PBS and cell associated SPIO particle content analyzed using the MPD as described below. Cell viability was measured using alamar Blue reagent (Invitrogen, Carlsbad, CA) and a fluorescence plate reader (Molecular Devices, Spectra Max Gemini XS) exactly as described by the reagent manufacturer. In our laboratory, the alamar blue assay is comparable to the MTT assay with the advantage of not being influenced by interference from nanoparticles [35] and data not shown.

#### Real time quantitative RT-PCR

The methods for primer selection, amplification and analysis are described in the in vivo methods section. The list of primers can be found in Additional file 11.

### ISDD simulation of in vitro SPIO dose-rate and cellular association

The accumulation of SPIO nanoparticles associated with C10 epithelial cells during in vitro exposures was modeled as a function of SPIO nanoparticle delivery rate and a term representing the fraction of particles delivered that remained associated with the cells after washing and harvest. The term represents the fraction of delivered material taken up by the cells and/or remains sufficiently adhered to the cells that it is not removed during washing. The fraction of material delivered to cells that remained associated with cells was fitted to the experimental data, and was time dependent. SPIO nanoparticle delivery to cells was calculated using the In Vitro Sedimentation Diffusion and Dosimetry (ISDD) model [16], as previously described. ISDD is a computational model of particle transport to cells in liquid cell culture systems. ISDD was previously validated for iron oxide particles. The particles, which were agglomerates, were modeled as with a fractal dimension of 2.1 and packing factor 0.637 [16]. The particle diameter was equal to the value reported by TEM (13 nm), and the agglomerate size was the value measured by DLS in the cell culture media (276 nm). The number of particles per agglomerate was calculated according to Sterling as described in Hinderliter et al. [16]. Other model parameters were: temperature, 310°K; media density, 1.0 g/cm^3^; media viscosity, 0.00074 Pa●s (reflecting the presence of serum proteins in the media), and a particle density of 5.2 g/cm^3^. The medial volume was 2.5 ml and the media height was 2.6 mm.

## Additional files

Additional file 1:
**Micrographs.** SEM (a) and TEM (b) images of as produced (Fe_3_O_4_) SPIO particles and a SEM image of the carboxylated SPIO particles. Electron microscopy was used to determine the primary particle size and the crystallinity of the particles. Additional file 2:
**Particle Characteristics.** Average hydrodynamic sizes, polydispersity and zeta potentials of Fe_3_O_4_ nanoparticles in water suspension at pH ~ 7.5 before and after carboxylation. Additional file 3:
**Spatial uniformity of SPIO inhalation exposure across tiers and ports of the carousel.**
Additional file 4:
**Tidal volume of mice prior to and during nanoparticle exposures.**
Additional file 5:
**Respiratory rate of mice prior to and during nanoparticle exposures.**
Additional file 6:
**Minute ventilation rate of mice prior to and during nanoparticle exposures.**
Additional file 7:
**Comparison of mouse and human lung dosimetry for SPIO particles regional deposited mass doses predicted by MPPD were similar for the nano and microscale particles.**
Additional file 8:
**A, B: Standard curves for SPIO nanoparticle content.** Standard curves were made in the appropriate biological matrix, for cell culture unexposed cells (A), and for mouse tissues (B). Additional file 9:
**Custom exposure system for generating SPIO nanoparticles and conducting animal exposures.**
Additional file 10:
**Regional surface areas of the mouse used to calculate deposited doses normalized to lung region surface area.**
Additional file 11:
**Primer sequences for target genes.**

## Abbreviations

ADME: Adsorption, distribution, metabolism and elimination
ANOVA: Analysis of variance
BMM: Bone marrow derived macrophage
CMD: Count median diameter
ECD: Electron capture detector
FDR: False discovery rate
GSD: Geometric standard deviation
HEPA: High efficiency particle air (filter)
ISDD: In vitro sedimentation diffusion and dosimetry model
MMD: Median mass diameter
MOUDI: Micro-orifice uniform deposit impactor
MPD: Magnetic particle detection
MPPD: Multipath particle deposition model
mRNAs: Messenger ribonucleic acid
MV: Minute volume
OP: Oropharyngeal
PCR: Polymerase chain reaction
PEL: Permissible Exposure Limit
OSHA: Occupational Safety and Health Administration
RH: Relative humidity
RNA: Ribonucleic Acid
RR: Respiratory rate
RSD: Root mean square difference
RT-PCR (qRT-PCR): Reverse transcriptase polymerase chain reaction
SEM: Scanning electron microscopy
SMPS: Scanning mobility particle sizer
SPIO: Super paramagnetic iron oxide
TEM: Transmission electron microscopy
TIM: Total inhaled mass
TV: Tidal volume
U.S. EPA: United States Environmental Protection Agency
