# Supplementary figures and images for: Comparative iron oxide nanoparticle cellular dosimetry and response in mice by the inhalation and liquid cell culture exposure routes
Source: Part Fibre Toxicol. 2014 Sep 30;11:46. doi: 10.1186/s12989-014-0046-4 (PMC4200214; doi:10.1186/s12989-014-0046-4)

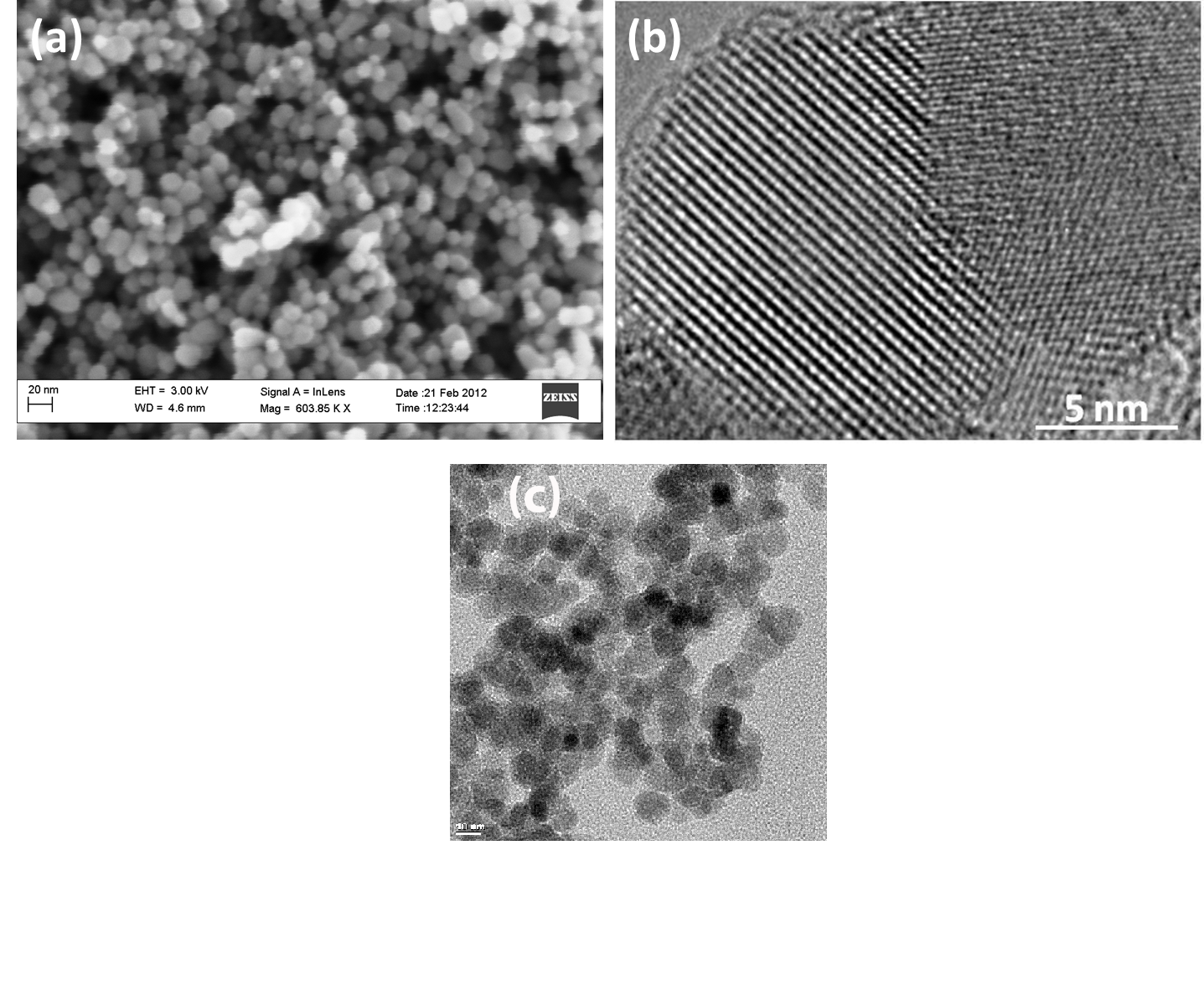

Supplement: Additional file 1: — Micrographs. SEM (a) and TEM (b) images of as produced (Fe3O4) SPIO particles and a SEM image of the carboxylated SPIO particles. Electron microscopy was used to determine the primary particle size and the crystallinity of the particles. [file 12989_2014_46_MOESM1_ESM.tiff]

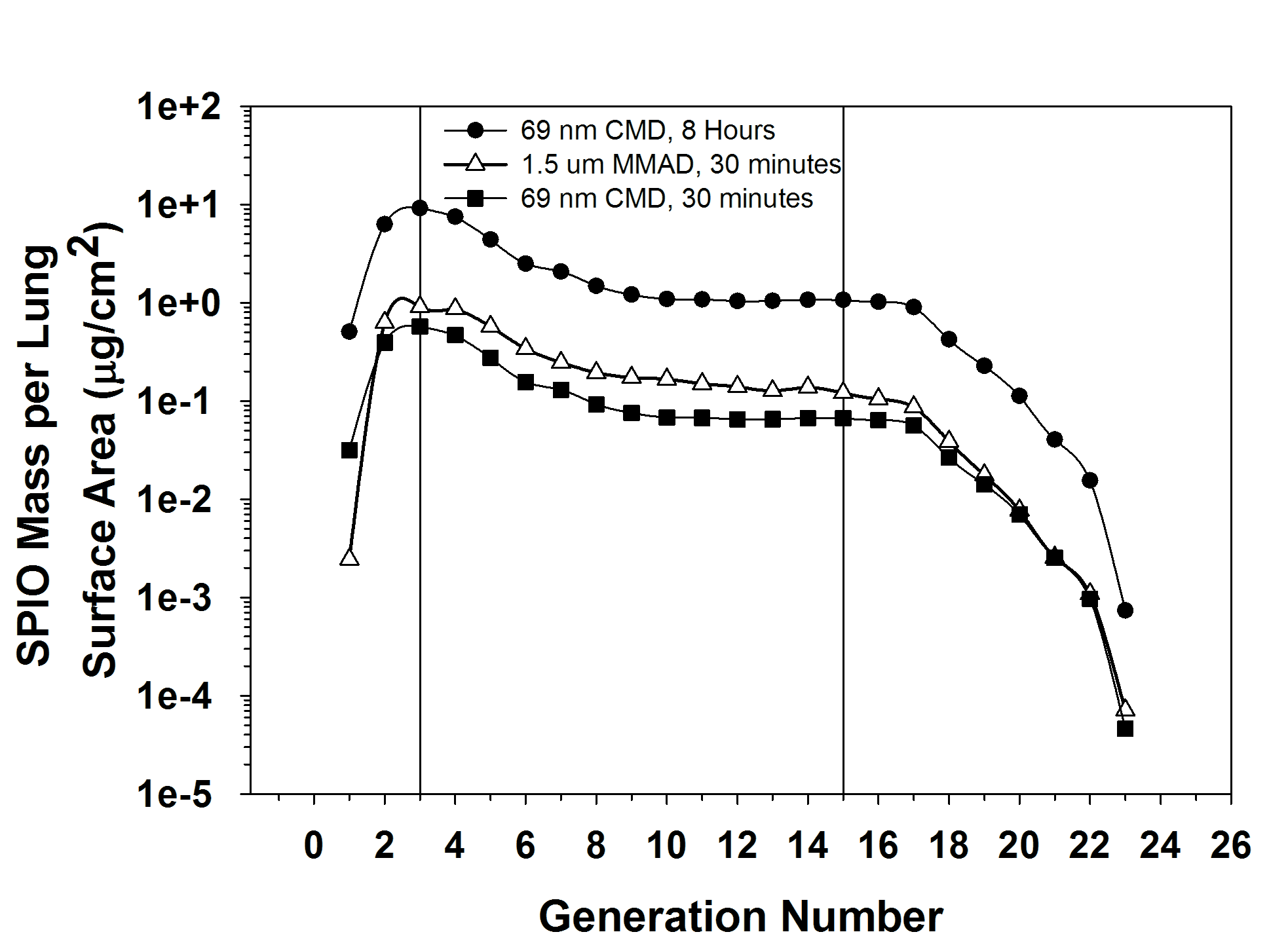

Supplement: Additional file 7: — Comparison of mouse and human lung dosimetry for SPIO particles regional deposited mass doses predicted by MPPD were similar for the nano and microscale particles. [file 12989_2014_46_MOESM7_ESM.tiff]

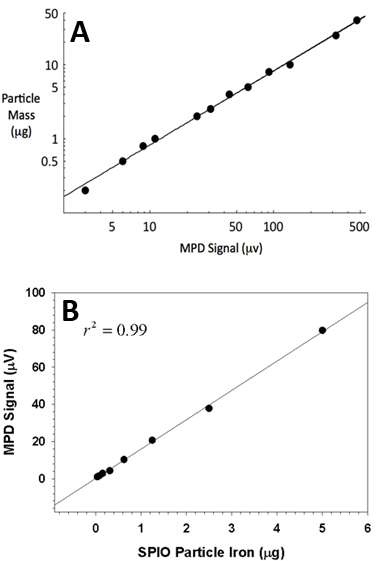

Supplement: Additional file 8: — A, B: Standard curves for SPIO nanoparticle content. Standard curves were made in the appropriate biological matrix, for cell culture unexposed cells (A), and for mouse tissues (B). [file 12989_2014_46_MOESM8_ESM.tiff]
