# Supplementary material for: Comparative iron oxide nanoparticle cellular dosimetry and response in mice by the inhalation and liquid cell culture exposure routes
Source: Part Fibre Toxicol. 2014 Sep 30;11:46. doi: 10.1186/s12989-014-0046-4 (PMC4200214; doi:10.1186/s12989-014-0046-4)
Supplement: Additional file 2: — Particle Characteristics. Average hydrodynamic sizes, polydispersity and zeta potentials of Fe3O4 nanoparticles in water suspension at pH ~ 7.5 before and after carboxylation. [file 12989_2014_46_MOESM2_ESM.docx]

**Additional file 2. Average hydrodynamic sizes, polydispersity and zeta potentials of Fe_3_O_4_ nanoparticles in water suspension at pH~7.5**

| Surface modification | *S*ize *(nm)* ± polydispersity *(%)* | Zeta potential (mV) |
| --- | --- | --- |
| Before | 144.8 ± 26.4 | -20.3 ± 2.8 |
| After | 56.2 ± 18.7 | -41.0 ± 3.7 |
