# Supplementary material for: Comparative iron oxide nanoparticle cellular dosimetry and response in mice by the inhalation and liquid cell culture exposure routes
Source: Part Fibre Toxicol. 2014 Sep 30;11:46. doi: 10.1186/s12989-014-0046-4 (PMC4200214; doi:10.1186/s12989-014-0046-4)
Supplement: Additional file 3: — Spatial uniformity of SPIO inhalation exposure across tiers and ports of the carousel. [file 12989_2014_46_MOESM3_ESM.docx]

**Additional file 3. Spatial uniformity of SPIO inhalation exposure across tiers and ports of the carousel.**

.

| **Tier (T) and Port (P) #** | **Aerosol Number Concentration^a^**  **(10^6^ particles/cm^3^)** |
| --- | --- |
| T2, P1 | 7.6 |
| T1, P1 | 8.2 |
| T1, P5 | 8.5 |
| T2, P3 | 8.4 |
| T2, P5 | 8.5 |
| T2, P1 | 8.4 |
| T2, P7 | 9.0 |
| T3, P1 | 8.6 |
| T3, P5 | 9.0 |
| T4, P1 | 8.7 |
| T4, P5 | 8.9 |
| T2, P1 | 9.4 |
| Between Tier Average (SD, RSD) | 8.6 (0.26, 3.1%) |
| Within Tier Average (SD, RSD) | 8.6 (0.28, 3.3%) |
| ^a^Particle numbers have been multiplied by 10^-6^. |  |
