# Supplementary material for: Comparative iron oxide nanoparticle cellular dosimetry and response in mice by the inhalation and liquid cell culture exposure routes
Source: Part Fibre Toxicol. 2014 Sep 30;11:46. doi: 10.1186/s12989-014-0046-4 (PMC4200214; doi:10.1186/s12989-014-0046-4)

**Additional file 5**: Respiratory rate was measured in 5 treated (Top Panel) and 5 control animals (Bottom Panel) for the initial 30 or 10 minutes of exposure and the final 30 minute of the 4 hour exposure using a whole body plethysmograph fitted to the exposure carousel.


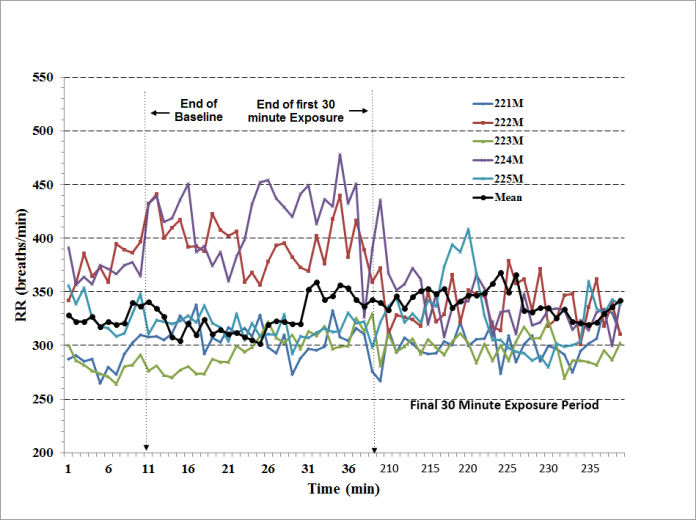


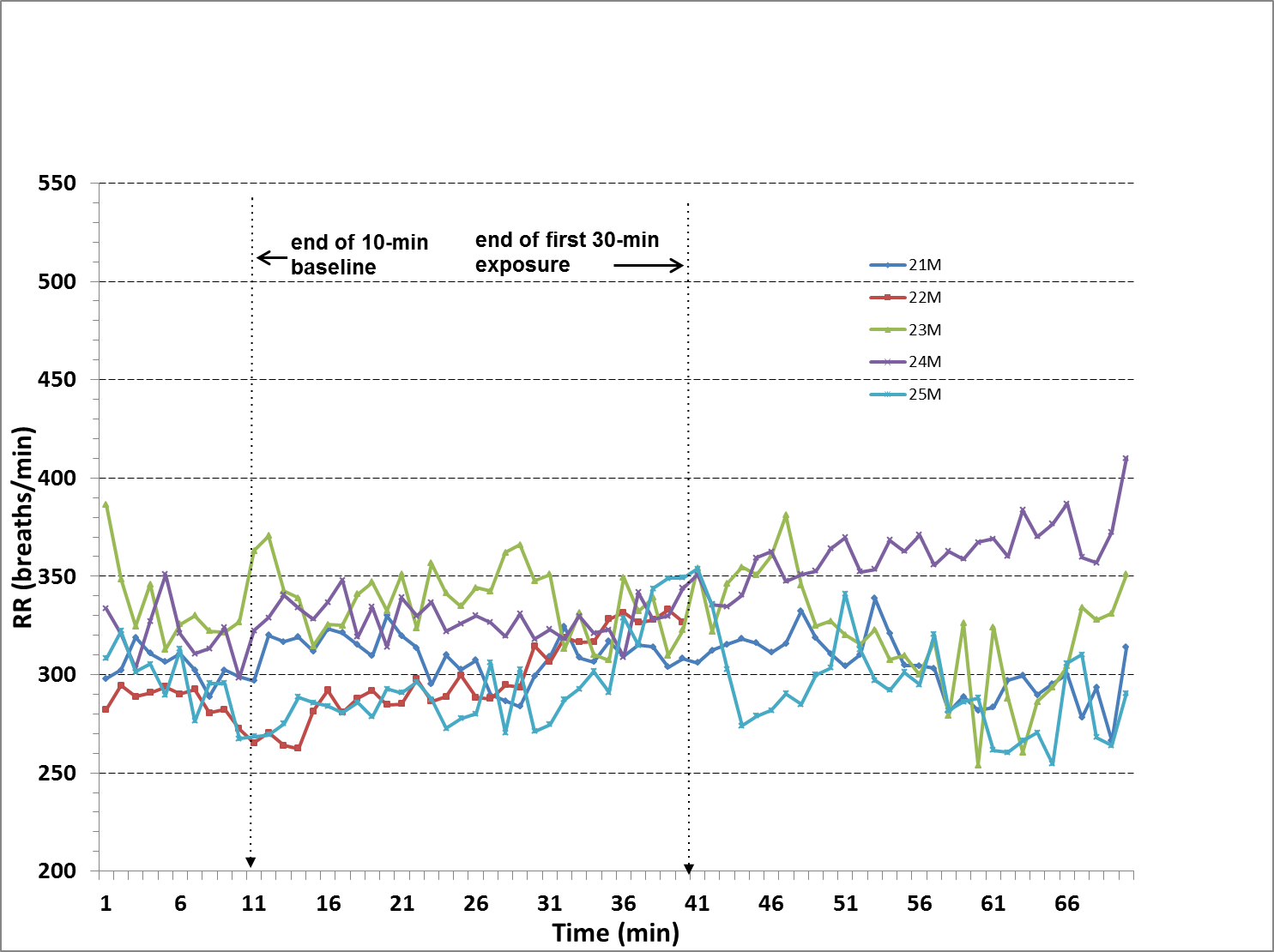

Supplement: Additional file 5: — Respiratory rate of mice prior to and during nanoparticle exposures. [file 12989_2014_46_MOESM5_ESM.docx]
