# Supplementary material for: Comparative iron oxide nanoparticle cellular dosimetry and response in mice by the inhalation and liquid cell culture exposure routes
Source: Part Fibre Toxicol. 2014 Sep 30;11:46. doi: 10.1186/s12989-014-0046-4 (PMC4200214; doi:10.1186/s12989-014-0046-4)

**Additional file 9:** General Schematics of the SPIO Nano-Aerosol Generation and Nose-Port Inhalation System. Sampling ports at each level of the carousel allowed constant monitoring of aerosol characteristics. A plethesmorgraph was used to measure respiratory parameters.


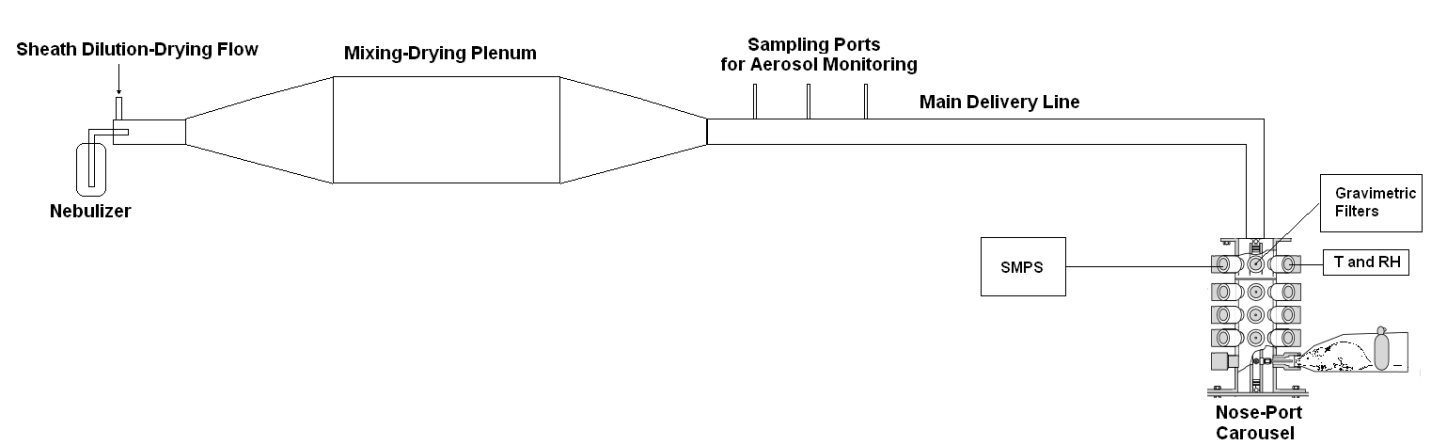

Supplement: Additional file 9: — Custom exposure system for generating SPIO nanoparticles and conducting animal exposures. [file 12989_2014_46_MOESM9_ESM.docx]
