# Supplementary material for: Comparative iron oxide nanoparticle cellular dosimetry and response in mice by the inhalation and liquid cell culture exposure routes
Source: Part Fibre Toxicol. 2014 Sep 30;11:46. doi: 10.1186/s12989-014-0046-4 (PMC4200214; doi:10.1186/s12989-014-0046-4)

**Additional file 10:** Regional surface areas of the mouse used to calculate deposited doses normalized to lung region surface area.


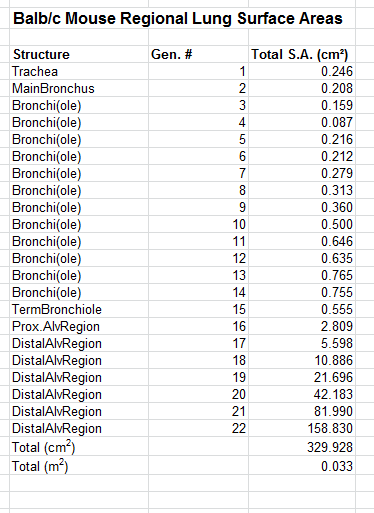

Supplement: Additional file 10: — Regional surface areas of the mouse used to calculate deposited doses normalized to lung region surface area. [file 12989_2014_46_MOESM10_ESM.docx]
