# Supplementary material for: Comparative iron oxide nanoparticle cellular dosimetry and response in mice by the inhalation and liquid cell culture exposure routes
Source: Part Fibre Toxicol. 2014 Sep 30;11:46. doi: 10.1186/s12989-014-0046-4 (PMC4200214; doi:10.1186/s12989-014-0046-4)
Supplement: Additional file 11: — Primer sequences for target genes. [file 12989_2014_46_MOESM11_ESM.docx]

**Additional file 11**: Primer sequence for genes used in this study

| **Gene name** | **Accession** | **Primer sequences** |
| --- | --- | --- |
| CXCL1 | NM_008176.3 | F: 5’-agactccagccacactccaa-3’  R: 5’-tgacagcgcagctcattg-3’ |
| CXCL2 | NM_009140.2 | F: 5’-aaaatcatccaaaagatactgaacaa-3’  R: 5’-ctttggttcttccgttgagg-3’ |
| CXCL3 | NM_203320.2 | F: 5’-ccccaggcttcagataatca-3’  R: 5’-tctgatttagaatgcaggtcctt-3’ |
| CCL3 | NM_011337.2 | F: 5’-tgcccttgctgttcttctct-3’  R: 5’-gtggaatcttccggctgtag-3’ |
| CXCL5 | NM_009141 | F: 5´ -cctggtccgggatcttgt- 3´  R: 5´ -catgaatggcgagatggaa- 3´ |
| CCL9 | NM_011338 | R: 5´ -accagtggtgggtgtacca- 3´  F: 5´ -catctctgaactctccgatcact- 3´ |
| CCL15 | NM_011339 | F: 5´-tgctcaaggctggtccat- 3´  R: 5´-gacatcgtagctcttgagtgtca- 3´ |
| GDF15 | NM_011819 | F: 5´ -gagctacggggtcgcttc- 3´  R: 5´ -gggaccccaatctcacct- 3´ |
| VNN1 | NM_011704 | F: 5´ -tggttgcgagataccataagc- 3´  R: 5´ -ctccatggggacattgaact- 3´ |
